# Which factors drive the choice of the French‐speaking Quebec population towards a COVID‐19 vaccination programme: A discrete‐choice experiment

**DOI:** 10.1111/hex.13963

**Published:** 2024-01-05

**Authors:** Gabin F. Morillon, Thomas G. Poder

**Affiliations:** ^1^ Montpellier Recherche en Économie University of Montpellier Avenue Raymond Dugrand Montpellier France; ^2^ Department of Management, Evaluation and Health Policy, School of Public Health University of Montreal Montreal Quebec Canada

**Keywords:** COVID‐19, discrete‐choice, health economics, hesitancy, preferences, Quebec, vaccine

## Abstract

**Objectives:**

The aims of this study were to elicit preferences about the coronavirus disease 2019 (COVID‐19) vaccine campaign in the general French‐speaking adult Quebec population and to highlight the characteristics of the vaccine campaign that were of major importance.

**Methods:**

A discrete‐choice experiment (DCE) was conducted between April and June 2021, in Quebec, Canada. A quota sampling method by age, gender and educational level was used to achieve a representative sample of the French‐speaking adult population. The choice‐based exercise was described by seven attributes within a vaccine campaign scenario. A mixed logit (MXL) model and a latent class logit (LCL) model were used to derive utility values. Age, gender, educational level, income and fear of COVID‐19 were included as independent variables in the LCL.

**Results:**

A total of 1883 respondents were included for analysis, yielding 22,586 choices. From these choices, 3425 (15.16%) were refusals. In addition, 1159 (61.55%) individuals always accepted any of the vaccination campaigns, while 92 individuals (4.89%) always refused vaccine alternatives. According to the MXL, relative weight importance of attributes was effectiveness (32.50%), risk of side effects (24.76%), level of scientific evidence (22.51%), number of shots (15.73%), priority population (3.60%), type of vaccine (0.61%), and vaccination location (0.28%). Four classes were derived from the LCL model and attributes were more or less important according to them. Class 1 (19.8%) was more concerned about the effectiveness (27.99%), safety (24.22%) and the number of shots (21.82%), class 2 (55.3%) wanted a highly effective vaccine (40.16%) and class 3 (17.6%) gave high value to the scientific evidence (42.00%). Class 4 preferences (7.4%) were more balanced, with each attribute having a relative weight ranging from 1.84% (type of vaccine) to 21.32% (risk of side effects). Membership posterior probabilities to latent classes were found to be predicted by individual factors such as gender, annual income or fear of COVID‐19.

**Conclusions:**

Vaccination acceptance relies on multiple factors. This study allowed assessment of vaccination‐specific issues through a choice‐based exercise and description of factors influencing this choice by segmenting the sample and drawing profiles of individuals. Moreover, besides effectiveness and safety, a major point of this study was to show the importance given by the general population to the level of scientific evidence surrounding vaccines.

**Patient or Public Contribution:**

A small group of citizens was involved in the conception, design and interpretation of data. Participants of the DCE were all from the general population.

## 1 INTRODUCTION

On 11 March 2020, the World Health Organization (WHO) characterized coronavirus disease 2019 (COVID‐19) as a pandemic.^1^ As of 11 May 2022, 15,201 deaths were reported in the province of Quebec, Canada, out of a total of 39,984 deaths in Canada.^2^ Since the beginning of the pandemic, mass vaccination has appeared to be the best way out of the crisis. However, an increase in vaccination hesitancy was already observed before the pandemic.^3^ It is therefore useful to examine the characteristics of vaccination programmes that determine the choice of vaccination. For instance, on 11 May 2022, 13.9% of 18–29‐year‐old and 14.0% of 30–49‐year‐old Quebec residents had not been adequately vaccinated (i.e., ‘proportion of the population who received two doses of vaccine or a single dose for people with a laboratory‐confirmed history of COVID‐19’).^4^

Our focus was on vaccination‐specific issues (e.g., introduction of a new vaccine, design of a vaccination programme, vaccination schedule, costs) as defined by the Vaccine Hesitancy Determinants Matrix developed by the SAGE Working Group on Vaccine Hesitancy.^5^ Because of the rapid development of the health situation at the time of the study, it remained difficult to provide an accurate picture of the status of the crisis.^1^ Vaccines were new and still in development, and the vaccination campaign and restriction policies were still evolving. This may have led to confusion, doubts and distrust of authorities in the general population.

Discrete‐choice experiment (DCE) is a stated preference method that aims to elicit preferences throughout a set of choice tasks based on a finite set of alternatives.^6^ Although hypothetical, these choices may reflect decisions made in real‐life settings. This method relies on the random utility theory^7^, ^8^ and on the ability of an individual to make choices between vaccine campaigns with the same characteristics (attributes) but different modalities (levels).

Several studies using conjoint analyses, such as choice‐, ranking‐ or rating‐based methods, have been conducted worldwide to explore COVID‐19 vaccination preferences (see Louviere et al.^9^ for a discussion about differences between conjoint analysis and DCE). Notable investigations have been carried out in China,^10^, ^11^, ^12^, ^13^, ^14^, ^15^ the United States,^13^, ^16^, ^17^, ^18^, ^19^, ^20^, ^21^ Canada,^22^ Australia^23^ and Europe.^24^, ^25^, ^26^, ^27^, ^28^ We also considered previous conjoint analysis on vaccination^29^, ^30^ and a systematic review.^31^ These studies concerned different populations (i.e., general population, students, healthcare workers) and focused on the vaccination campaign and factors associated with vaccination acceptance. Based on a non‐exhaustive literature review, we identified commonly used attributes in vaccine conjoint experiments that we described here in three dimensions: vaccine‐specific attributes, vaccination campaign attributes and institutional attributes. Table 1 provides a summary of the above‐mentioned studies.

**Table 1:** DCE COVID‐19 vaccination literature.

Reference^a^, ^b^	Country	Year of survey	Attribute														
			Mild side effects	Major side effects	Vaccination effectiveness	Protection duration	Number of doses	Vaccination location	Type of vaccine	Brand/Manufacturer	Origin of vaccine	Vaccine recommendation	Vaccine approval	Waiting time to get vaccinated	Cost/price/financial incentive	Family/friends vaccinated or intending to be vaccinated	Other attributes^c^
[10]	China	2020	**X**		**X**	**X**	**X**				**X**				**X**		
[11]	China	n.r.		**X**	**X**	**X**	**X**	**X**							**X** d	**X**	
[12]	China (Hong Kong)	2021	**X**		**X**	**X**	**X**				**X**				**X** e		
[13]	ChinaUnited States	2021	**X**		**X**				**X**						**X**		Time for the vaccine to start working
[14]	China	2020	**X**		**X**		**X**	**X**			**X**				**X**		Health Code colour^f^
[15]	China (Hong Kong)	2021		**X**	**X**			**X**		**X**						**X**	Quarantine for vaccinated travellers
[16]	United States	2020	**X**	**X**	**X**	**X**					**X**	**X**	**X**				
[17]	United States	2020	**X**		**X**		**X**		**X**		**X**						Months spent in development
[18]	United States	2020	**X**	**X**	**X**												
[19]	United States	2020		**X**	**X**	**X**		**X**									Proof of vaccination
[20]	United States	2021					**X**	**X**						**X** g		**X**	Vaccination appointment scheduling, vaccination enforcement and vaccine frequency
[21]	United States	n.r.	**X**		**X**					**X**			**X**		**X**		
[22]	Canada (Quebec)	2020	**X**		**X**	**X**					**X**	**X**		**X**			Priority population to receive the vaccine
[23]	Australia	2020	**X**		**X**	**X**			**X**						**X**	**X**	Mode of administration
[24]	United Kingdom	2020			**X**		**X**	**X**				**X**					Coverage in the media
[25]	France	2020		**X**	**X**			**X**			**X**						
[27]	France	2020/2021			**X**	**X**						**X** h					Indirect protectionVaccine safety^i^
[28]	Netherlands	2020	**X**	**X**	**X**							**X**		**X**			
[29]	Netherlands	2013		**X** j	**X**										**X**		Media coverage about the vaccine
[30]	China (Hong Kong)	2018	**X**		**X**			**X**								**X**	Programme duration, vaccination arrangement procedure and vaccination service hours

Abbreviations: COVID‐19, coronavirus disease 2019; DCE, discrete‐choice experiment; n.r., not reported.

^a^ Only studies on vaccination uptake are reported (e.g., studies on policies or population prioritization are not reported).

^b^ For more details about the definition of attributes and their levels, see references.

^c^ Some attributes were rarely used in the reported studies.

^d^ The authors included in this attribute a compulsory vaccination dimension: Access to vaccine (levels: free and voluntary; free and compulsory; chargeable and voluntary).

^e^ The out‐of‐pocket payment included all doses required to get fully vaccinated.

^f^ Individuals who refused the vaccine were attributed a different health code and ‘forego their rights to access […] public spaces’.

^g^ Waiting time to get vaccinated is at the waiting room.

^h^ This attribute includes a level about who is already vaccinated: 80% of healthcare workers in other European countries have been vaccinated.

^i^ Vaccine safety was defined by the authors as ‘[…] different types of information regarding the safety of the proposed vaccine’.

^j^ The authors provided a description of ‘safety of the vaccine’: ‘Long term severe side effects (death, life‐threatening events, hospitalization, severe or permanent handicap, or side effects leading to birth defects to an unborn fetus)’ and added that ‘before the start of the choice tasks, respondents were informed that on the short term, vaccinations resulted in mild side effects only’.

The realm of vaccine preferences and priorities encompasses a spectrum of attributes intrinsic to the vaccines themselves. Central among these, the effectiveness of the vaccine (i.e., decrease in the probability to develop a symptomatic form of the disease within a real‐world setting) stands as a pivotal influencer of individual decisions to pursue vaccination. Equally critical is the consideration of potential side effects, with the perceived risk of adverse reactions weighing heavily on the decision‐making process. The type of vaccine also plays a discernible role, as individuals may show preferences for traditional formulations or novel messenger RNA (mRNA)‐based approaches. Furthermore, the duration of protection that the vaccine promises holds sway, with individuals factoring this temporal aspect into their vaccination choices. The number of doses required and the resulting convenience or complexity of the dosing schedule further contribute to shaping these preferences.

In addition to vaccine‐specific attributes, characteristics of the vaccination campaign itself exert significant influence. Financial implications, notably the cost associated with vaccination, play a central role in decision‐making. Individuals often consider the monetary impact alongside other attributes when determining their willingness to be vaccinated. Convenience and ease of reaching vaccination sites and manageable waiting times also feature prominently, reflecting practical concerns that can sway individuals' preferences.

Beyond individual attributes and campaign logistics, the recognition of official institutions profoundly affects vaccination choices. Regulatory approvals and endorsements by health authorities contribute to the perceived credibility and safety of the vaccine. Individuals are more likely to trust and opt for vaccines that have garnered institutional backing. The reputation and history of the vaccine manufacturer further influence decisions. Recognizing the significance of these institutional factors is essential in shaping vaccination strategies that resonate with diverse populations and engender higher levels of acceptance.

To our knowledge, this study is the first DCE to include the evidence level of scientific publication about the vaccine as a characteristic of the COVID‐19 vaccination campaign. Moreover, beyond effectiveness and safety, it explored other concerns such as the type of vaccine (including mRNA), the number of shots required (up to three shots) or the priority population to get vaccinated (including primary and secondary teachers, which was a matter of debate in the media at the time of the survey). In this study, we aimed to elicit preferences of the adult Quebec general population about the COVID‐19 vaccine campaign using a DCE. This was done to better understand vaccine behaviours and to highlight the characteristics of the vaccine campaign that are of major importance.

## 2 METHODS

### 2.1 Survey design

An online survey was conducted between 6 April and 29 June 2021 by Dynata Inc. during the COVID‐19 vaccination campaign in the province of Quebec, Canada. We targeted the French‐speaking adult Quebec population and used a quota sampling method by age, gender and educational level as described by the national statistics.^32^ Using these quota restrictions, panellists from Dynata Inc. were randomly invited to participate. At the time of the survey, four vaccines were authorized by the Canadian government (i.e., AstraZeneca, Pfizer‐BioNTech, Moderna, Janssen),^33^ with different effectiveness (i.e., from 65% to 95%, approximately), side effects, type of technology (i.e., viral vector, mRNA, subunit and plant‐based) or number of doses required. The Novavax's Nuvaxovid COVID‐19 vaccine was authorized but deployed on 17 February 2022, in adults aged over 18, which was several months after the study. At the launch of the survey, 19.3% of the entire Quebec population received at least one dose and only 0.9% was adequately vaccinated.^4^ At the end of the survey, these numbers were equal to 71.2% and 29.1%, respectively.

There is no consensus about the sample size in the literature about DCE. Some authors recommended a minimum of 10–20 responses per choice task to obtain efficient coefficients.^34^, ^35^ Then, a minimum of 600 respondents was required knowing that we had 360 choice tasks and that each respondent answered 12 choice tasks (i.e., $360\times \frac{20}{12}=600$). However, to obtain a representative sample of the adult French‐speaking general population in Quebec, a sample size of  1067 individuals was required.* The survey was divided into several parts: sociodemographic and socioeconomic variables, DCE and other measures about vaccination and COVID‐19.

### 2.2 Sociodemographic variables and survey instruments

Usual sociodemographic characteristics (e.g., age, gender, marital status, educational level, income) of the respondents were obtained and questions related to health condition (e.g., serious illness, health problems affecting quality of life, health status) were asked. Three scales about satisfaction with health, satisfaction with life and willingness to take risk, all scales ranging from 0 to 10, and several questions about COVID‐19 experiences such as past infection (i.e., has had COVID‐19 in the past and when), financial losses and vaccination (i.e., intention to get vaccinated and side effects if vaccinated) were also included.

A set of survey instruments was included in the online questionnaire. We assessed vaccine trust and hesitancy,^36^ perception of the risks relative to COVID‐19, fear of COVID‐19 (FCV‐19S)^37^, ^38^ and individuals' sense of coherence (SOC‐3).^39^, ^40^ Details about the variables' coding and the measurements are provided in Supporting Information S1: File 1.

### 2.3 DCE design

The DCE design followed the recommendations in the field.^41^, ^42^, ^43^, ^44^ A mixed‐method study was performed. First, a nonexhaustive literature review was conducted to collect information about vaccination campaigns that are of importance in the choice to get vaccinated and to create a first list of the attributes and their levels. Vaccine effectiveness and side effects were found to be always analysed in the vaccine choice‐based literature, and number of shots, vaccination location and type of vaccine were commonly used in the current literature about COVID‐19 vaccination.^10^, ^11^, ^12^, ^13^, ^14^, ^15^, ^16^, ^17^, ^18^, ^19^, ^20^, ^21^, ^22^, ^23^, ^24^, ^25^, ^26^, ^27^, ^28^, ^29^, ^30^, ^45^ This explains our choice to include them in our DCE. Note that the choice for the type of vaccine was based on a WHO series of articles about COVID‐19 vaccination.^46^ The levels selected for effectiveness and side effects were based on scientific data published by the Canadian public health agency at the start of the survey for the vaccines in use in Canada.^47^ Two other attributes were also selected, considering two debates in Quebec in early 2021: the level of evidence of data associated with vaccines and the population to be prioritized. For the level of evidence, which can be considered as a level of trust in the characteristics of the vaccine, we chose an attribute easily recognized by the population: vaccine whose results were published in a scientific journal (national or international). It is expected that people will have higher confidence in international publications, in that it will increase their trust in what is stated about the characteristics of the vaccine. In the priority population attribute, the level ‘primary and secondary teachers’ was selected according to a Quebec debate at the time of the survey,^48^ whereas the other level for ‘vulnerable population with a loss of autonomy’ was found to be generally accepted. Experts in public health (n=2) and health economics (n=1), as well as a few citizens (n=3), were consulted face to face, via phone‐ or web‐based interviews, to get their opinions about the literature review results and to provide further information and refinements about the attributes and the levels to be considered. In brief, this led to seven attributes with three to six levels each. At each step of the DCE design, the same experts and citizens were solicited to provide their inputs about the credibility, relevance and univocity of the attributes and levels. All of them provided extremely significant and relevant information to state the most important attributes and levels, considering the context of the COVID‐19 vaccination campaign at that time in Quebec. The number of interviews was limited considering the convergent information provided. The final selection is provided in Table 2.

**Table 2:** Attributes and levels of the discrete‐choice experiment.

Attributes and levels
Type of vaccine
Inactivated vaccine (i.e., contains inactivated or dead virus)
Live‐attenuated vaccine (i.e., contains a weakened version of the virus)
Viral vector vaccine (i.e., contains a harmless virus that harbours some of the virus's genetic material)
Subunit vaccine (i.e., contains specific parts of the virus [e.g., proteins])
DNA vaccine (i.e., contains part of the genetic material of the virus, namely, DNA)
Messenger RNA vaccine (i.e., contains part of the genetic material of the virus, the messenger RNA)
Vaccine effectiveness
95%
85%
75%
60%
Side effects of the vaccine
None
One in 1000 chances of having a minor effect (e.g., redness, fevers)
One in 10,000 chances of having a major effect (e.g., hospitalization, persistent disabilities)
Vaccination location
A care centre (e.g., hospital, long‐term care homes, community health centre)
A sports centre, an exhibition centre
A drugstore
Priority population to receive the vaccine
No priority population
Primary and secondary teachers
Vulnerable people with great loss of autonomy (e.g., CHSLD, RI‐RTF)
Number of shots to be received
One shot
Two shots
Three shots
Vaccine whose results have been published in a scientific journal
Unpublished results
Results published in a national journal
Results published in an international journal

Since our DCE included seven attributes with three to six levels each, a total of 5832 scenarios (i.e., $6\times 4\times 3^{5}$) and 34,006,392 choice cards (i.e., 5832×(5832−1)) could be generated. An orthogonal selection procedure was performed in Lighthouse Studio 9.7.2 to produce 300 choice tasks. The D‐efficiency value was 0.97. Thirty blocks of 10 choice tasks were randomly generated and randomly assigned to each respondent. In each block, a rationality/dominant test (i.e., one scenario that was considered to completely dominate the other one in choice task 1) and a temporal consistency test (i.e., choice task 2 was repeated in choice task 12) were added. Thus, each respondent faced 12 choice tasks and this yielded a total of 360 choice tasks.

**Figure 1:**
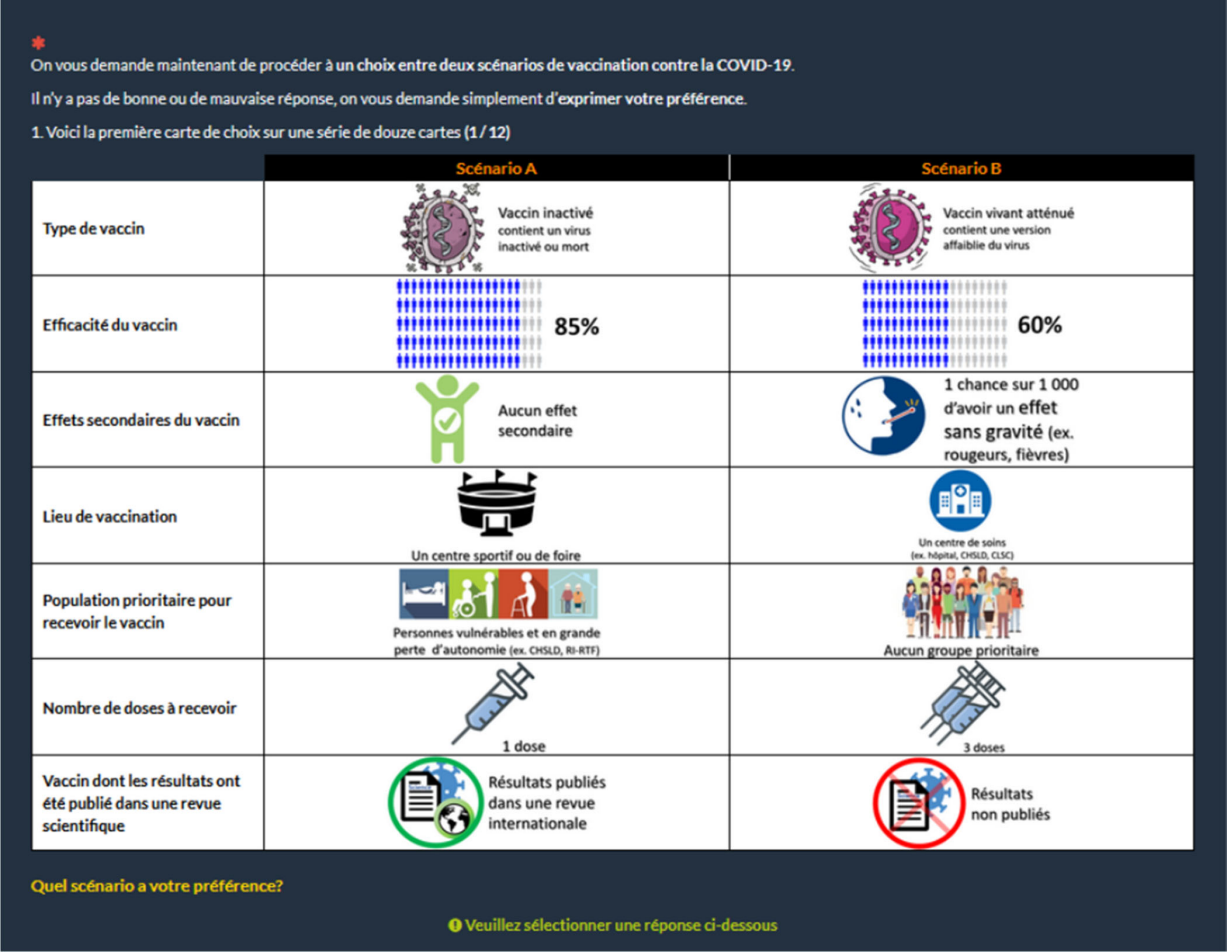
Illustration of a choice task (in French). Respondents were given the option of choosing an opt‐out alternative, specified below the choice card.

Each choice task included two unlabelled vaccine alternatives (Figure 1). To add realism to the exercise, we included an opt‐out alternative (i.e., respondents were given the possibility not to choose a vaccine).^49^, ^50^ Each level was dummy‐coded: the level took the value of 1 when it was described in the scenario and 0 otherwise (see Hu et al.^51^ for a discussion about dummy and effects coding). Considering the panel nature of the data, the alternative specific constant (ASC) was invariant across individuals and choice tasks and coded as a triad of (1, 1, 0) (alternative A, alternative B and opt out). Although this approach may entail some loss of generalizability, it facilitates a more streamlined model and simpler estimation. For a more comprehensive explanation, please refer to Campbell and Erdem,^52^ section 2.2.1. See Supporting Information S1: File 2 for an illustration of the data set including an output choice.

A set of debrief questions was used after the exercise to exclude observations and for a better interpretation of the results.^53^ After each choice task of the DCE, respondents rated their choice certainty answer between 0 (not certain at all) and 10 (absolutely certain). All choice certainty questions were summed to obtain a score (ranging from 0 to 120). Then, we included several questions such as the exercise difficulty, the number of attributes considered and the quality of the answers. We also asked the respondents if they answered to the best of their ability and if they had been annoyed or irritated by the choice tasks. Finally, respondents were asked to rank the attributes according to their importance. It allowed us to assess if the personal ranking was in accordance with the choice exercises. However, a ranking exercise does not incorporate a renunciation (i.e., a choice) as a DCE does.

Before conducting analyses, a set of exclusion criteria based on the debrief questions was applied:

1. Respondent chose vaccine opt‐out at most nine times over 12 in less than 1 min.
2. Respondent always chose option A or always chose option B.
3. Respondent provided poor or very poor quality of answers.
4. Respondent stated not having answered to the best of his or her ability.

### 2.4 Choice models

To estimate the representative utility levels,^54^ a mixed logit (MXL) (also known as random‐parameter logit)^55^, ^56^ with 1000 Halton draws and a latent class logit (LCL)^57^, ^58^ were performed. The MXL estimates the parameters of a specified distribution. Thus, for a normal distribution, the model estimates a coefficient for the mean and another coefficient for the standard deviation for each level. A significant standard deviation coefficient for a given level indicates heterogeneity in the preferences for this level. Moreover, it alleviates the independence of irrelevant alternatives hypotheses that states that the relative probability of selecting alternatives should not change if we introduce or eliminate another alternative.^54^ The LCL is a class membership model that predicts discrete shares of individuals presenting similar preference patterns (intraclass) that are heterogeneous from one group to another one (interclass). Both models allowed us to consider unobserved preference heterogeneity in vaccination choice.

Preference heterogeneity was also deepened by specifying independent variables in the class membership model. Doing so allowed us to point out some observable heterogeneity. Individuals' characteristics (invariant within individuals) considered were age, gender (male), educational level (no university degree), annual income (less than 85,000 CAD) and Fear‐of‐COVID19 Scale (score equal to 24 or less over 35). All were found to be good predictors of vaccine acceptance in Quebec at the beginning of the pandemic.^22^, ^59^ Indeed, females and older individuals, with higher levels of education, a high income and strong fear of COVID‐19 were more likely to trust and accept the COVID‐19 vaccine.

For clarity, the models are presented according to the best hypothetical vaccination campaign (i.e., preferred levels by respondent): a viral vector vaccine, effective at 95%, with no side effect, administered in a drugstore, with no priority population, with one injection required and whose results were published in an international journal. Models were assessed on the basis of Akaike and Bayesian information criteria (AIC, BIC) and the log‐likelihood function. The number of latent classes was chosen according to the same criteria and the relative likelihood.† ^60^ Results were expressed as raw utility values and in terms of conditional relative importance weights.

### 2.5 Descriptive analysis methods

The sample is described according to the nature of the variables (i.e., mean, standard error, median and proportion [column frequencies]). Relevant tests were performed (i.e., one‐way analysis of variance, Kruskal–Wallis *H*‐test, Bartlett's test for equality of variances, Fisher's exact test and the *χ* ^2^ test of independence). A p value <.05 was considered as statistically significant and all p values were reported.

## 3 RESULTS

### 3.1 Sample

Of 4321 individuals solicited, 2550 (i.e., 59.01%) started the survey and 2037 (i.e., 47.14%) completed the DCE. Among these 2037 individuals, we excluded a few because they responded to the 12 choice tasks in less than 1 min and chose 75% of the time or less (i.e., at most nine refusals out of 12) to refuse the vaccine (*n* = 90), always chose option A (*n* = 22) or option B (*n* = 6), provided poor (*n* = 52) or very poor (*n* = 8) quality of answers or because they stated not having answered to the best of their ability (*n* = 4). A total of 154 individuals (i.e., 7.56%) were excluded from the analysis, yielding a full sample of 1883 individuals. See Supporting Information S1: File 3 for details about excluded individuals.

This form of participant selection was used to present the main results, while other alternatives of participant selection are presented in Supporting Information S1: Files 4 and 5 as sensibility analyses. Thus, the same specification was followed: (1) with excluded respondents (*n* = 154); (2) without respondents who failed the rationality test or the temporal consistency test (*n* = 521); (3) only with respondents who declared having been vaccinated against COVID‐19 (*n* = 814) and (4) without respondents who declared the choice exercise as being ‘hard’ or ‘very hard’ (*n* = 90).

### 3.2 Descriptive analysis of the sample

After applying exclusion criteria, the sample was representative of the general French‐speaking adult population by age and gender but was unbalanced according to the educational level (Table 3). The sample used was made up of almost as many women as men (male/female ratio equal to 0.96), had a mean age of 51.32 years (±18.28) was mainly married or living with a partner (57.25%) and was employed (46.68%) or retired (36.91%). About 35.85% were highly educated. The mean annual household income was 67,752 CAD (±42,312). Less than 75% lived in an urban area, the majority in the Greater Montreal area (43.55%).

**Table 3:** Sample sociodemographic characteristics.

Sociodemographic characteristics		
	Sample (*n* = 1883)	General adult population
Gender		
Male	48.86%	49.40%
Female	51.09%	50.60%
Male/female ratio	0.96	0.98
Age (years)		
Mean (SD)	51.32 (18.28)	49.80
Range	18–94	
Marital status		
Single	29.79%	29.40%
Married/living with a partner	57.25%	56.30%
Divorced/separated	8.92%	8.60%
Widowed	4.04%	5.70%
Occupational status		
Employed	46.68%	59.50%
Retired	36.91%	27.4%^a^
At home	4.41%	
Student	5.20%	3.30%
Unemployed	4.62%	7.20%
Sick and parental leave	2.12%	2.50%
Educational level^b^		
Secondary or less and Diploma of professional studies	35.85%	23.5%^c^
College and CEGEP	28.20%	48.70%
Baccalaureate, Master and PhD	35.85%	27.80%
Annual household income (CAD)		
Mean (SD)	67,752.26 (42,312.08)	61,061
Range	2500–165,000	
Type of residence (urban)	72.17%	80.60%
Live in the Grand Montréal	43.55%	48.00%

^a^ For occupational status, 27.4% were ‘other’ (probably included those who had retired and were at home).

^b^ See International Standard Classification of Education (UNESCO): http://uis.unesco.org/sites/default/files/documents/international-standard-classification-of-education-isced-2011-en.pdf.

^c^ Among the active population, Annuaire québécois des statistiques du travail 2008–2018, vol.5, Institut de la statistique du Québec, see https://statistique.quebec.ca/en/fichier/annuaire-quebecois-des-statistiques-du-travail-portrait-des-principaux-indicateurs-du-marche-et-des-conditions-de-travail-2008-2018-volume-15.pdf.

About 11.05% experienced fairly significant or very significant financial losses due to COVID‐19 and 45.25% continued to work during the lockdown (Table 4). Among them, 18.08% were healthcare workers. About 4.20% had been sick with COVID‐19, whereas 10.36% and 16.36% had a family member or a relative, respectively, who had been sick with COVID‐19 in the past months. The FCV‐19S was about 16.82/35 (±6.31, *α* = .901). More than one‐tenth (11.42%) had not been vaccinated against COVID‐19 and did not plan to get vaccinated. Among individuals already vaccinated, 67.20% reported no side effects. Pain or itching were the most commonly reported side effects (27.15%). The COVID‐19 Risk Beliefs Score was 5.45/9 (±2.21, *α* = .613), indicating relatively low negative belief in the risks associated with the pandemic and vaccination. The Vaccine Trust Score was 5.16/6 (±1.47, *α* = .808) and the Vaccine Hesitancy Score was 9.21/32 (±6.27, *α* = .794), indicating high trust and relatively low hesitation in vaccination.

**Table 4:** COVID‐19 experiences, COVID‐19 vaccination and vaccination perception.

COVID‐19 experiences, COVID‐19 vaccination and vaccination perception	
Financial losses due to COVID‐19	
No financial loss	58.42%
Small financial losses	30.54%
Fairly significant financial losses	9.13%
Very significant financial losses	1.91%
Continued to work during the COVID‐19 lockdown (from March to May 2020)	45.25%
Work status during the COVID‐19 lockdown	
Essential worker (health worker)	18.08%
Essential worker (other profession)	50.59%
Nonessential worker	31.34%
Have you or a relative been sick with COVID‐19 in the past few months?	
Yourself	4.20%
A family member	10.36%
A relative	16.36%
COVID‐19 vaccination intention	
Already vaccinated	43.23%
Not vaccinated but plan to be vaccinated	45.35%
Not vaccinated and do not plan to be vaccinated	11.42%
Side effects following the injection	
No side effect	67.20%
Pain or itching	27.15%
Fever	4.67%
Redness at the injection site	5.04%
Have been hospitalized	0.12%
Fear of COVID‐19 Score (ranging from 7 to 35)^a^	
Mean (SD)	16.86 (6.31)
Range	7–35
COVID‐19 Risk Beliefs Score^a^	
Mean (SD)	5.45 (2.21)
Range	0–9
Vaccine Trust Score (ranging from 0 to 6)^a^	
Mean (SD)	5.16 (1.47)
Range	0–6
Vaccine Hesitancy Score (ranging from 0 to 32)^a^	
Mean (SD)	9.21 (6.27)
Range	0–32

Abbreviation: COVID‐19, coronavirus disease 2019.

^a^ See Supporting Information S1: File 1 for details.

The median response time to the DCE was about 4:10 min, and the Choice Certainty Score was 95.45/120 (±20.66) (Table 5). More than half found the exercise ‘easy’ (35.74%) or ‘very easy’ (17.79%) and considered three or more attributes in their choices (53.64%). About 46.70% provided ‘good’ quality of answers, and almost one‐fifth stated that they were annoyed or irritated by the exercise. About 4.89% (*n* = 92) never chose one or the other vaccine campaign in the DCE (Supporting Information S1: File 6) and 61.55% (*n* = 1159) never refused the vaccine campaign. Almost 6% (*n* = 104) and 25% (*n* = 455) failed the rationality test and the temporal consistency test, respectively.

**Table 5:** DCE debrief questions.

DCE debrief questions	
Response time to the DCE (hh:mm:ss)	
Mean (SD)	05:18 (05:57)
Median	04:10
Range	00:36–01:22:06
Choice Certainty Score (ranging from 0 to 120)^a^	
Mean (SD)	95.45 (20.66)
Range	0–120
Choice exercise difficulty	
Very easy	17.79%
Easy	35.74%
Neutral	41.69%
Hard	4.62%
Very hard	0.16%
Number of dimensions considered	
1	6.27%
2	28.50%
3 or more	53.64%
Not sure	11.59%
Quality of responses	
Very good	38.58%
Good	46.70%
Average	14.72%
Have been annoyed	19.18%
Always refused one or the other vaccine campaign	4.89%
Never refused one or the other vaccine campaign	61.55%
Failed the rationality test	5.52%
Failed the temporal consistency test	24.16%

Abbreviation: DCE, discrete‐choice experiment.

^a^ The Choice Certainty Score corresponds to the sum of each certitude questions ‘Are you sure of your choice?’ scaled between 0 and 10. The score ranges from 0 to 120.

### 3.3 Preference analysis

A total of 22,596 choices were recorded, with a vaccine refusal rate of 15.16% (equating to 3425 choices declined). On average, the vaccine type, except for the live‐attenuated vaccine (*p* = .039), which demonstrated notable disutility, along with the vaccine location, had no significant impact on individuals' willingness to opt in (Table 6). However, individuals showed a greater likelihood of opting out in scenarios where the vaccine effectiveness was below 95%, accompanied by potential side effects, administered through multiple shots and without results published in an international journal. Furthermore, a higher likelihood of opting out was observed if the vaccine was specifically designated for teachers in primary and secondary schools (*p* < .001).

**Table 6:** Multinomial mixed‐logit model.

Attribute	Mixed logit model									
	Mean					Standard deviation				
	Coefficient	Standard error	p Value	Confidence interval		Coefficient	Standard error	p Value	Confidence interval	
ASC	6.294***	0.175	<.001	5.951	6.638	4.332	0.165	<.001	4.010	4.655
Type of vaccine										
Viral vector vaccine	(reference)					(reference)				
mRNA vaccine	0.035	0.055	.528	−0.073	0.143	0.393**	0.126	.002	0.146	0.640
Subunit vaccine	−0.082	0.057	.148	−0.193	0.029	0.442***	0.120	<.001	0.206	0.677
DNA vaccine	−0.094	0.055	.088	−0.203	0.014	0.224	0.164	.172	−0.097	0.545
Inactivated vaccine	0.038	0.055	.492	−0.070	0.146	0.597***	0.097	<.001	0.408	0.786
Live‐attenuated vaccine	−0.116*	0.056	.039	−0.225	−0.006	0.398**	0.116	.001	0.171	0.625
Vaccine effectiveness										
95%	(reference)					(reference)				
85%	−0.656***	0.041	<.001	−0.737	−0.575	0.153	0.130	.239	−0.102	0.408
75%	−1.380***	0.048	<.001	−1.474	−1.286	0.393**	0.114	.001	0.169	0.617
60%	−2.563***	0.069	<.001	−2.698	−2.427	1.379***	0.066	<.001	1.250	1.508
Side effects of the vaccine										
None	(reference)					(reference)				
One in 1000 chance of having a minor effect	−0.536***	0.035	<.001	−0.606	−0.467	0.369***	0.088	<.001	0.197	0.541
One in 10000 chance of having a major effect	−1.414***	0.055	<.001	−1.521	−1.306	1.411***	0.060	<.001	1.293	1.528
Vaccination location										
A drugstore	(reference)									
A care centre	0.003	0.037	.936	−0.069	0.075	0.170	0.173	.327	−0.170	0.510
A sports centre, an exhibition centre	−0.023	0.037	.532	−0.094	0.049	0.306**	0.099	.002	0.112	0.500
Priority population to receive the vaccine										
No priority population	(reference)					(reference)				
Vulnerable people with great loss of autonomy	−0.034	0.037	.350	−0.106	0.038	0.268	0.119	.025	0.034	0.501
Primary and secondary teachers	−0.251***	0.039	<0.001	−0.327	−0.174	0.397***	0.072	<.001	0.257	0.537
Number of shots to be received										
One shot	(reference)					(reference)				
Two shots	−0.228***	0.036	<.001	−0.298	−0.157	0.352***	0.096	<.001	0.163	0.540
Three shots	−0.928***	0.045	<.001	−1.015	−0.840	0.948***	0.054	<.001	0.841	1.054
Vaccine whose results have been published in a scientific journal										
Results published in an international journal	(reference)					(reference)				
Results published in a national journal	−0.148***	0.034	<.001	−0.215	−0.081	−0.037	0.109	.732	−0.252	0.177
Unpublished results	−1.461***	0.054	<.001	−1.566	−1.355	1.325***	0.057	<.001	1.212	1.437
Relative importance weights of attributes										
Type of vaccine	0.61%									
Vaccine effectiveness	32.50%									
Side effects of the vaccine	24.76%									
Vaccination location	0.28%									
Priority population to receive the vaccine	3.60%									
Number of shots to be received	15.73%									
Vaccine whose results have been published in a scientific journal	22.51%									
Observations	67,788									
*χ* ^2^	9531.710									
Log likelihood	−14,522.790									
Null log likelihood	−19,288.640									
AIC	29,121.583									
BIC	29,256.941									

*Note*: 1000 Halton draws specified.

Abbreviations: AIC, Akaike information criterion; ASC, alternative specific constant; BIC, Bayesian information criterion.

* *p* < .05

** *p* < .01

*** *p* < .001.

While nearly all individuals showed a preference for a 95% effectiveness rate (with only 3.16% favouring a 60% efficacy level), approximately 15.81% expressed a preference for a one in 10,000 chance of encountering a major side effect. Additionally, 44.89% indicated a preference for prioritizing vulnerable individuals experiencing significant loss of autonomy, while 16.39% favoured a vaccination campaign comprising three shots. Furthermore, 13.51% expressed a preference for a vaccine whose results had not been published (Supporting Information S1: File 7).

For the sake of clarity, results can also be expressed in terms of conditional relative importance weights. Considering the MXL model, effectiveness (32.50%), side effects (24.76%) and publication of the results (22.51%) were the most important attributes, whereas priority population, type of vaccine and vaccine location were of very low importance (3.60%, 0.61% and 0.28%, respectively). The number of shots to be received was of moderate importance relative to the other attributes (i.e., 15.73%). However, because of the preference heterogeneity, these results were nuanced by the LCL model. According to the information criteria and the relative likelihood, four latent classes were derived (Table 7 and Supporting Information S1: File 8).

**Table 7:** Latent class logit model (four classes).

Attribute	Latent class logit																			
	Class 1					Class 2					Class 3					Class 4				
	Coefficient	Standard error	*p* Value	95% Confidence interval		Coefficient	Standard error	*p* Value	95% Confidence interval		Coefficient	Standard error	*p* Value	95% Confidence interval		Coefficient	Standard error	*p* Value	95% Confidence interval	
ASC	2.084***	0.137	<.001	1.816	2.352	5.542***	0.156	<.001	5.236	5.847	5.276***	0.250	<.001	4.786	5.765	−3.052***	0.579	<.001	−4.186	−1.918
Type of vaccine																				
Viral vector vaccine	(reference)																			
mRNA vaccine	−0.068	0.104	.512	−0.273	0.136	0.172**	0.053	.001	0.069	0.276	−0.467**	0.153	.002	−0.767	−0.167	0.482	0.596	.419	−0.687	1.651
Subunit vaccine	−0.111	0.100	.265	−0.307	0.085	0.049	0.053	.356	−0.055	0.152	−0.420**	0.159	.008	−0.731	−0.109	−0.054	0.524	.918	−1.080	0.973
DNA vaccine	−0.142	0.100	.153	−0.338	0.053	0.024	0.052	.648	−0.078	0.125	−0.381*	0.153	.013	−0.680	−0.081	−1.249	0.797	.117	−2.811	0.312
Inactivated vaccine	0.099	0.097	.310	−0.092	0.289	0.014	0.053	.786	−0.089	0.118	−0.441**	0.150	.003	−0.735	−0.146	0.962*	0.478	.044	0.025	1.899
Live‐attenuated vaccine	−0.029	0.098	.768	−0.222	0.164	−0.077	0.053	.148	−0.182	0.027	−0.379*	0.162	.019	−0.696	−0.062	0.239	0.511	.640	−0.763	1.241
Vaccine effectiveness		Vaccine effectiveness																		
95%	(reference)																			
85%	−0.467***	0.071	<.001	−0.605	−0.329	−0.526***	0.041	<.001	−0.606	−0.446	−0.513***	0.122	<.001	−0.752	−0.275	−0.226	0.335	.500	−0.882	0.430
75%	−1.028***	0.080	<.001	−1.186	−0.871	−1.032***	0.044	<.001	−1.118	−0.946	−1.037***	0.143	<.001	−1.317	−0.756	−0.650	0.431	.132	−1.496	0.195
60%	−1.979***	0.102	<.001	−2.178	−1.780	−1.784***	0.051	<.001	−1.884	−1.684	−1.548***	0.163	<.001	−1.868	−1.229	−0.710	0.399	.075	−1.492	0.071
Side effects of the vaccine																				
None	(reference)																			
One in 1000 chance of having a minor effect	−0.416***	0.062	<.001	−0.538	−0.294	−0.318***	0.033	<.001	−0.384	−0.253	−0.713***	0.100	<.001	−0.909	−0.518	−0.716	0.367	.051	−1.436	0.003
One in 10,000 chance of having a major effect	−1.264***	0.080	<.001	−1.420	−1.108	−0.791***	0.040	<.001	−0.869	−0.713	−1.474***	0.123	<.001	−1.716	−1.232	−0.766*	0.388	.048	−1.526	−0.007
Vaccination location																				
A drugstore	(reference)																			
A care centre	−0.034	0.068	.610	−0.167	0.098	−0.011	0.034	.757	−0.077	0.056	0.217*	0.095	.022	0.031	0.403	0.249	0.372	.503	−0.480	0.978
A sports centre, an exhibition centre	−0.010	0.066	.878	−0.139	0.119	−0.010	0.034	.777	−0.077	0.058	−0.024	0.096	.804	−0.211	0.164	0.312	0.335	.352	−0.345	0.970
Priority population to receive the vaccine																				
No priority population	(reference)																			
Vulnerable people with great loss of autonomy	−0.137*	0.068	.042	−0.270	−0.005	−0.089**	0.034	.009	−0.157	−0.022	0.078	0.101	.440	−0.120	0.277	0.672	0.381	.078	−0.074	1.418
Primary and secondary teachers	−0.226**	0.068	.001	−0.360	−0.092	−0.232***	0.035	<.001	−0.301	−0.163	−0.212*	0.100	.035	−0.408	−0.015	0.423	0.384	.272	−0.331	1.176
Number of shots to be received																				
One shot	(reference)																			
Two shots	−0.420***	0.065	<.001	−0.547	−0.293	−0.116**	0.034	.001	−0.184	−0.049	−0.059	0.099	.554	−0.253	0.136	−0.451	0.333	.176	−1.102	0.201
Three shots	−1.001***	0.072	<.001	−1.143	−0.860	−0.568***	0.035	<.001	−0.637	−0.499	−0.501***	0.112	<.001	−0.721	−0.282	−0.959**	0.387	.013	−1.718	−0.200
Vaccine whose results have been published in a scientific journal																				
Results published in an international journal	(reference)																			
Results published in a national journal	−0.113	0.062	.068	−0.234	0.008	−0.065	0.034	.056	−0.132	0.002	−0.108	0.078	.171	−0.261	0.046	−0.805*	0.366	.028	−1.523	−0.086
Unpublished results	−1.089***	0.083	<.001	−1.251	−0.927	−0.429***	0.042	<.001	−0.511	−0.347	−3.359***	0.216	<.001	−3.782	−2.935	−0.512	0.314	.103	−1.128	0.104
Class share (membership probability)	0.655**	0.237	.006	0.189	1.120	0.958***	0.214	<.001	0.539	1.377	0.746**	0.253	.003	0.251	1.241	(reference)				
Mean class share (probability of choice)	0.198	0.371				0.553	0.445				0.176	0.311				0.074	0.257			
Age (ref: aged 18–59)	0.441*	0.213	.038	0.024	0.857	−0.044	0.190	.816	−0.415	0.327	0.041	0.226	.856	−0.403	0.485	(reference)				
Gender (ref.: male)	0.585*	0.258	.024	0.078	1.092	0.669**	0.235	.004	0.209	1.130	1.241***	0.262	<.001	0.727	1.755					
Educational level (ref.: no higher degree)	0.477	0.388	.219	−0.283	1.237	0.729*	0.353	.039	0.036	1.421	0.516	0.407	.204	−0.281	1.314					
Income (ref.: less than 85,000 CAD)	0.507*	0.257	.049	0.003	1.011	0.507*	0.234	.030	0.049	0.965	0.894**	0.263	.001	0.378	1.410					
Fear‐of‐COVID19 Scale (ref.: score equal to 24 or less over 35)	0.202	0.206	.326	−0.202	0.607	1.312***	0.177	<.001	0.964	1.659	−0.145	0.232	.533	−0.599	0.310					
Relative importance weights of attributes																				
Type of vaccine	0.68%					0.68%					4.94%					1.84%				
Vaccine effectiveness	27.99%					40.16%					18.75%					12.17%				
Side effects of the vaccine	24.22%					24.00%					24.05%					21.32%				
Vaccination location	0.71%					0.48%					2.32%					8.67%				
Priority population to receive the vaccine	5.41%					7.06%					1.32%					16.19%				
Number of shots to be received	21.82%					15.91%					6.62%					20.82%				
Vaccine whose results have been published in a scientific journal	19.17%					11.71%					42.00%					18.99%				
Observations	67,788																			
Log likelihood	−14,945.08																			
AIC	30,078.17																			
BIC	30,935.84																			

Abbreviations: ASC, alternative specific constant; AIC, Akaike information criterion; BIC, Bayesian information criterion; COVID‐19, coronavirus disease 2019; ref, reference.

* *p* < .05

** *p* < .01

*** *p* < .001.

Beginning with the ASC, the results derived from the MXL analysis revealed a significant inclination among individuals to favourably consider a COVID‐19 vaccination campaign (*p* < .001) based on the reference levels. Classes 1, 2 and 3 within the LCL demonstrated positive and noteworthy coefficients (*p* < .001), signifying a greater likelihood for individuals to select a vaccine campaign aligned with the specified reference levels rather than opting out. In contrast, class 4 showed a significantly negative ASC coefficient (*p* < .001), indicating a preference among individuals to opt out rather than get vaccinated against COVID‐19 based on the reference levels. Moreover, individuals in class 4 showed an even stronger propensity to opt out when the vaccine involved three shots, more side effects and for which results had been exclusively published in a national journal.

For classes 1, 2 and 3 (i.e., classes more likely to opt in), the levels of effectiveness, side effects and number of shots, as well as a vaccine for which results were unpublished, and priority for primary and secondary teachers, were significant (at least at p<.05) and consistent in the decrement, except for class 3, where individuals were more likely to opt out if the vaccine comprises three shots. Regarding the vaccine type, class 2 was more likely to prefer an mRNA vaccine (p=.001), while class 3 was more likely to opt in only if the vaccine was a viral vector vaccine. Prioritizing vulnerable people also resulted in significant disutility for classes 1 and 2 (p=.042 and p=.009, respectively). Individuals were somewhat likely to be indifferent about the vaccination location, except for those in class 3, who were more likely to opt in if the vaccine is administered in a care centre (p=.022). Finally, individuals were more likely to be female and to have a higher annual income than those in class 4. Respondents in class 1 were also more likely to be older (p=.038), while in class 2, they were more likely to have higher educational qualifications (p=.039) and greater fear of COVID‐19 (p<.001).

In terms of conditional relative importance weights, class 1 assigned higher importance to effectiveness (27.99%), safety (24.22%) and the number of shots (21.82%), class 2 assigned higher importance to effectiveness (40.16%) and class 3 assigned higher importance to scientific evidence (42.00%) and safety (24.05%). Conditional relative importance weights were well balanced in class 4, except for the vaccination location (8.67%) and the type of vaccine (1.84%). Graphical representations of raw utility values and statistics of class membership posterior probabilities are given in Supporting Information S1: Files 9 and 10.

## 4 DISCUSSION

### 4.1 Summary

In this study, we conducted a DCE and found that effectiveness, safety and publication of scientific results (i.e., level of evidence) were of major importance in COVID‐19 vaccination acceptance in Quebec, Canada. Furthermore, the general adult Quebec population showed relative indifference to the type of vaccine and the vaccine location, and moderate disutility in vaccine involving three shots and weak disutility if primary and secondary teachers were prioritized. However, these patterns were nuanced by a latent class analysis resulting in different preference behaviours.

Most of the attributes used in the DCE were part of the vaccine‐specific factors (i.e., ‘directly related to vaccine or vaccination’). As in the recent literature about COVID‐19 vaccine conjoint analysis and, more generally speaking, vaccine conjoint analysis, our results indicated that vaccine effectiveness and safety were of utmost importance, contributing together to half or more in explaining the choice of disutility respondents. Such a result was also found in a previous study in Quebec before the start of the vaccination campaign.^22^ However, other attributes of importance should be investigated in the specific context of the COVID‐19 pandemic.

The type of vaccine was not found to be significant in the MXL model, except for the live‐attenuated vaccine, which led to a low level of disutility (p=.039). Other studies found similar results: Motta^17^ found an indifference between weakened (i.e., live‐attenuated) and mRNA vaccine types, whereas Liu et al. (2021)^13^ found an indifference between mRNA and viral vector vaccine types but disutility for inactivated vaccine in the US, and disutility for inactivated vaccine and a preference for the viral vector vaccine in China compared to mRNA vaccine. However, the type of vaccine was generally of relatively low importance in choices (4.36% in the United States and 9.35% in China^13^ compared to 0.61% in our study). Regarding vaccine types available in Canada (i.e., viral vector, mRNA and subunit), a general indifference about the vaccine type and the absence of a live‐attenuated vaccine are likely to be positive points.

Although the vaccine location was not significant in the MXL model, the LCL model showed that some individuals preferred somewhat to get vaccinated in a care centre instead of a sport centre, which was in line with several studies^11^, ^14^, ^19^, ^24^, ^25^ where individuals preferred to get vaccinated in a medical setting (e.g., general hospital, local GP surgery, drugstore) instead of a nonmedical setting (e.g., mobile vaccination unit, mass vaccination centre). However, the study by Borriello et al.^23^ showed more complex behaviours about the vaccine location regarding the subgroup analysis, with individuals preferring a vaccination performed at a doctor's surgery and others preferring vaccine administration at a pharmacy.

Prioritizing healthcare workers or no population was previously found to induce low disutility in the general adult Quebec population but was of low importance in choices (2.11%)^22^, while we found that the same population was now indifferent between prioritizing no population and vulnerable people but was somewhat against prioritizing teachers in primary and secondary schools (p<.001). This attribute was also of low importance in choices in our study (3.60%).

About the number of doses required, our results matched with the majority of studies in the scientific literature,^10^, ^11^, ^14^, ^17^, ^20^, ^24^ that is, more doses required induced higher disutility, especially when more than two doses are needed. However, an exception is the study by Li et al.^12^ showing that university students in Hong Kong preferred more than one dose. This trend in preferences could be problematic since most developed countries have already implemented a third dose, or even a fourth dose, and others are planned.

The risk/benefit tradeoff of the vaccine is also documented by the scientific literature. While the level of scientific evidence is somewhat omitted in the COVID‐19 vaccine choice‐based literature, this attribute was found to be of utmost importance in our study. Indeed, it was the third most important attribute behind effectiveness and risk of side effects. Therefore, good communication and access about safety and effectiveness data, as well as other information, from reliable studies seem very necessary. For instance, this is done by the Canadian Government^33^ or the Centers for Disease Control and Prevention (CDC)^61^, which provide reliable and updated data and information in English or French about the trend of the pandemic, the vaccination schedule, the side effects reported or explanations about vaccine types. We also found that this attribute was relatively more important than who recommends the vaccine according to a previous study conducted in Quebec in fall 2020 (just before the vaccination campaign started).^22^ As described by Dubé et al.,^62^ a vaccine programme should have multiple components and, therefore, there is a need for a good understanding of the context (e.g., sociodemographic characteristics of the vulnerable population, barriers, determinants of vaccination). Subsequent investigations could delve into the individual and group influences within the French‐speaking Quebec population.

Of 1883 respondents, only 4.89% (*n* = 92) refused the 24 vaccination alternatives (i.e., 12 choice cards with two options and an opt‐out) and 61.55% (*n* = 1159) always accepted one or the other alternative. This represented a substantial decrease in the number of vaccine refusals, where, of over 1599 respondents recruited 6 months ago with the same method, 7.44% always refused the vaccination alternatives and 37.71% always accepted the vaccination.^22^ Such a result may be explained by better knowledge and confidence in the COVID‐19 vaccination programme a few months after its launch. Overall, our study is in line with previous research and enhances and refines it by incorporating novel attributes, such as the type of vaccine and the number of doses to be administered. Additionally, we explore new levels of attributes, particularly concerning the priority population, providing valuable insights to build upon the existing knowledge.

### 4.2 Study limitations

A first limitation of our study was its cross‐sectional character, because of which data refer to only one period. We explained preferences about the structure of the COVID‐19 vaccine campaign; however, given the evolution of the virus spread, scientific knowledge since early 2020 and the strong uncertainty, preferences may change, and individuals may adopt coping strategies. Thus, this study needs to be considered within its context in spring 2021 and into the global corpus about COVID‐19 vaccine preferences.

Another limitation is the cognitive burden posed by the choice‐based exercise and the number of attributes. To assess this potential burden, we used several debrief questions such as exercise difficulty, the number of attributes considered or feeling of annoyance (Table 5). The results of these debrief questions led us to be confident about the quality of the data. The online nature of the survey also allowed us to determine the response time to each choice task. The median times showed a real learning process between the first and the last choice task (Supporting Information S1: File 11).

While some studies included the price^10^, ^12^, ^13^, ^21^, ^23^, ^29^ or the brand^15^, ^21^ of the vaccine, COVID‐19 vaccines are free for all Canadian residents^33^, which may have created confusion in the choice of vaccination, whereas including the brand could have generated unrealistic scenarios in addition to do not being reflective of the reality in Canada, where the population was generally not offered the choice of the vaccine. As a disadvantage, not having included the price in the DCE did not allow us to calculate the willingness to pay for the vaccination as it was done in some other studies.

A potential limitation was associated with the fact that effectiveness per se was not defined when described to participants. Vaccine effectiveness is defined as a decrease in the probability of developing a symptomatic form of the disease within a real‐world context (in contrast to efficacy, which is defined within a controlled clinical trial framework).^63^ Effectiveness could also refer to a severe form of the disease. This can create confusion in the understanding of effectiveness among the general population. In Quebec, effectiveness of vaccine was presented as the probability of not developing symptoms that will require hospitalization. However, so as not to pose an additional cognitive burden on respondents, we decided not to define this and to let the respondents interpret the term effectiveness on their own. This may have created a bias, but also reduced the amount of information provided. We thus had a trade‐off between essential information (e.g., definition of the type of vaccines) to allow respondents to answer efficiently. Furthermore, although several authors integrated protection duration as an attribute in their experiments, we chose not to include it—either as an attribute or in the definition of effectiveness (Figure 1). A previous study that we conducted in Quebec in 2020 revealed that protection duration was rated among the most crucial characteristics for the general adult population.^22^ The absence of a clear definition regarding effectiveness and protection duration might increase the unobserved part of the utility function of respondents, potentially diminishing the informative value derived from the representative utility function.†

We chose not to categorize the risk of side effects based on risk and severity, opting instead to define risk within the probabilities of 0, 1/1000 for minor side effects and 1/10,000 for major side effects, while a broader definition of side effects may still hold significance for the population. Although we focused on a single attribute for side effects, this approach facilitated the exploration of alternative dimensions while maintaining an appropriate attribute count and consistent results.

In the random utility theory, preferences are considered to be complete, monotonic and continuous. Thus, nonsignificant coefficients of an attribute, such as the type of vaccine, reflect indifference towards this attribute. That is, individuals should be able to consider all the given information. However, they may apply heuristics in their decisions, which violates the hypothesis. Individuals can ignore one or more attributes to reduce the cognitive burden. This is called attribute nonattendance (ANA). To deal with ANA, some authors developed models such as the endogenous attribute attendance model^64^ or the equality‐constrained latent class model.^65^, ^66^ ANA analysis in vaccine choice‐based exercises could be an avenue for research in the future.

### 4.3 Policy implications

The emphasis on the importance of scientific evidence in vaccine acceptance underscores the need for health authorities to prioritize evidence‐based approaches when developing vaccination campaigns. Recognizing the public's strong regard for scientific validation could lead to the implementation of more transparent and well‐informed vaccination strategies. Public health authorities should ensure that their vaccination campaigns are rooted in credible research and open communication about the scientific underpinnings of vaccine safety and effectiveness. They can leverage this insight to tailor their communication strategies, formulating messages that address these concerns and convey the level of scientific support for vaccination, which could enhance public trust and willingness to engage in vaccination efforts. Skilful political communication could help alleviate vaccine hesitancy and promote a more positive response to vaccination campaigns. The idea that comprehending vaccination behaviours and drivers of acceptance is critical extends beyond the current COVID‐19 context. This suggests that authorities should invest in long‐term health crisis preparedness and response strategies. Future health crises may necessitate similar preference studies to inform effective crisis management. By incorporating insights from preference studies into public health policy frameworks, authorities could enhance their response to health emergencies while ensuring public cooperation and adherence to recommended measures. Acknowledging the public's desire to navigate crises successfully and trusting their capacity to grasp scientific insights and stay well informed can serve as a foundation for future health crisis preparedness efforts.

## 5 CONCLUSION

Through exploration of preferences within a COVID‐19 vaccination campaign, we have outlined vaccination‐specific issues. Beyond assessing effectiveness and safety, a central focus of this study was to underscore the significant value attributed by the general population to the degree of scientific evidence supporting the vaccines. Consequently, this attribute should be incorporated into future research endeavours and integrated into the strategic framework of vaccination campaigns. We posit that studies of this nature, including our own, which delve into preferences surrounding COVID‐19 vaccines, play a vital role in not only effectively managing the current pandemic but also in better preparing for potential future health crises.

## AUTHOR CONTRIBUTIONS

**Gabin F. Morillon**: Writing—original draft; writing—review and editing; formal analysis; data curation; visualization; methodology. **Thomas G. Poder**: Conceptualization; investigation; funding acquisition; writing—original draft; methodology; validation; visualization; writing—review and editing; formal analysis; project administration; data curation; supervision. Thomas G. Poder secured funding and supervised data collection. Both authors contributed to the design, analysis and drafting of the manuscript.

## CONFLICT OF INTEREST STATEMENT

The authors declare no conflict of interest.

## ETHICS STATEMENT

This study was approved by the ethics committee of the CIUSSS de l'Est de l'île de Montréal under number 2021‐2385. Subjects indicated their consent by clicking on the start button at the end of the explanatory letter. All questionnaires were completed anonymously.

## Supporting information

Supporting information.

## Data Availability

The data that support the findings of this study are available from the corresponding author upon reasonable request.

## Footnotes

* We used the general formula for sample calculation, that is, $n=\frac{\frac{z^{2}p(1-p)}{e^{2}}}{1+\left(\frac{z^{2}p\left(1-p\right)}{e^{2}N}\right)}$, with n being the sample size, N the population size (6.5 million), e the error margin (3%) and z the statistical value (1.96 with a 95% confidence interval) (see https://www.surveymonkey.com/mp/sample-size-calculator).

^†^
Using the Akaike information criterion, the relative likelihood is given by: $\mathrm{RL}=e^{\frac{\mathrm{AI}C_{\min}-\mathrm{AI}C_{q}}{2}}$, with $\mathrm{AI}C_{\min}$ being the minimum AIC.

^†^
An individual n, n=1,…,N, faces j alternatives, j=1,…,J, during t scenarios, t=1,…,T, and gain utility from his choice defined by, $U_{\mathrm{njt}}=V_{\mathrm{njt}}+\epsilon_{\mathrm{njt}}$, with V being the representative utility function defined by the attributes and their levels and ϵ being a random term that can be explained by the omission of attributes, unobserved taste variations or measured errors (e.g., definition of the attributes, complexity of the exercise, number of scenarios).
