# Supplementary material for: Which factors drive the choice of the French‐speaking Quebec population towards a COVID‐19 vaccination programme: A discrete‐choice experiment
Source: Health Expect. 2024 Jan 5;27(1):e13963. doi: 10.1111/hex.13963 (PMC10767688; doi:10.1111/hex.13963)
Supplement: Supplementary file 1 — Supporting information. [file HEX-27-e13963-s001.docx]

[Summary 1](#_Toc153536135)

[Supplementary File 1 – Psychometric scales 2](#_Toc153536136)

[Supplementary File 2 – Illustration of the choice-based exercise data structure 3](#_Toc153536137)

[Supplementary File 3 – Details of excluded individuals 4](#_Toc153536138)

[Supplementary File 4 – Multinomial mixed logit model sensitivity analyses 10](#_Toc153536139)

[Supplementary File 5 - Respondent’s personal ranking of attributes 11](#_Toc153536140)

[Supplementary File 6 – Details of “anti-vaccine” individuals (refused 100% of the times vaccines alternatives) 12](#_Toc153536141)

[Supplementary File 7 – Multinomial Mixed Logit model interpretation 17](#_Toc153536142)

[Supplementary File 8 - Latent Class Logit information criteria 18](#_Toc153536143)

[Supplementary File 9 – Raw utility values by model (Multinomial Mixed Logit and Latent Class Logit) 19](#_Toc153536144)

[Supplementary File 10 – Statistics of class membership posterior probabilities derived from the Latent Class Logit model 20](#_Toc153536145)

[Supplementary File 11 - Mean and median response time to the 12 choice tasks 21](#_Toc153536146)

[References 22](#_Toc153536147)

# **Summary**

Those supplementary files provide several data and analyses about the survey.

We present details of the psychometrics questionnaires whether validated or not (i.e., Sense of Coherence 3-item, Fear-of-COVID19 Scale, Vaccine Trust Scale, Vaccine Hesitancy Scale, and COVID-19 Risk Beliefs Scale) used in the survey.

We present details about all statistics (sociodemographic, health, COVID-19, vaccination, DCE related statistics, and so on).

We also provide supplementary Multinomial Mixed Logit models as sensibility analysis in addition to the model presented into the manuscript.

Moreover, we included several data and graphs (e.g., raw utility values, response times to the choice-based exercise) for illustration and interpretation.

For more details about the questionnaire, the DCE design or the statistical methods, please see the “Methodology” section of the manuscript.

For further information or if you wish to report any mistake, please contact the corresponding author.

# **Supplementary File 1** – Psychometric scales

***COVID-19 risk perception***. We used three statements from “strongly disagree” to “strongly agree” to assess the risk beliefs in COVID-19 pandemic and vaccination (*“I am worried about the risk of side effects from the COVID-19 vaccine.”*, *“I believe the risks associated with the COVID-19 pandemic are generally exaggerated.”*, and *“I believe the risks associated with the COVID-19 pandemic are lower than the risks of side effects from the COVID-19 vaccine.”*). The three statements were scored with a 4-point Likert scale ranging from “strongly disagree” (0 or 3) to “strongly agree” (3 or 0) and were summed to obtain a score ranging from 0 (lowest risk perception) to 9 (highest risk perception).

***Fear of COVID-19***. We used the Fear-of-COVID19 Scale (FCV-19S) developed by Ahorsu et al. (2020)^1^ and adapted in Canadian-French by Attieh et al. (2022)^2^. The FCV-19S is a validated 7-item scale ranging from 7 (lowest fear) to 35 (highest fear), each item being assessed with a 5-point Likert scale ranging from “strongly disagree” (1) to “strongly agree” (5). Items are:

- *I am most afraid of coronavirus-19.*
- *It makes me uncomfortable to think about coronavirus-19.*
- *My hands become clammy when I think about coronavirus-19.*
- *I am afraid of losing my life because of coronavirus-19.*
- *When watching news and stories about coronavirus-19 on social media, I become nervous or anxious.*
- *I cannot sleep because I’m worrying about getting coronavirus-19.*
- *My heart races or palpitates when I think about getting coronavirus-19.*

***Sense-of-Coherence***. The Sense-of-Coherence 3-item (SOC-3) questionnaire^3,4^ was used to assess an individual’s capacity to deal with comprehensibility, manageability, and meaningfulness in a salutogenic conceptual framework. The SOC-3 ranges from 0 (lowest sense of coherence) to 6 (highest sense of coherence). Items are:

- *Do you usually see a solution to problems and difficulties that other people find hopeless?*
- *Do you usually feel that your daily life is a source of personal satisfaction?*
- *Do you usually feel that the things that happen to you in your daily life are hard to understand? (reverse)*

***Vaccine trust*.** A set of five questions was used to assess vaccination trust: importance of the vaccination (1. *“Do you consider vaccination to be important for the health of the population?”*), trust in health institutions, doctor, and the pharmaceutical industry (2. *“Do you trust Quebec's health institutions to recommend an effective and safe vaccine?”*, 3. *“Do you trust your doctor to recommend an effective and safe vaccine?”*, and 4. *“Do you trust the pharmaceutical industry to produce an effective and safe vaccine?”*), and vaccination situation (5. *“Regarding vaccination, which of the following best describes your situation: I accept most or all of the vaccines, I accept some of vaccines, I refuse most or all of the vaccines recommended to me”*). The first four were binary coded whereas the latter was scored from 0 (refuse vaccination) to 2 (accept vaccination). We summed each question to obtain a vaccine trust score from 0 (lowest trust) to 6 (highest trust).

***Vaccine hesitancy***. Vaccine hesitancy was assessed using a modified version of the Vaccine Hesitancy Scale (VHS) developed by Shapiro et al. (2018)^5^. Our version consisted in 8 items with a 5-point Likert scale, from “strongly disagree” (0 or 4) to “strongly agree” (4 or 0), and the score ranged from 0 (no hesitancy) to 32 (highest hesitancy). Items are:

- *I feel sufficiently informed about vaccination.*
- *I consider that there are health risks if my vaccination is delayed.*
- *I have already hesitated to get vaccinated.*
- *I consider that we are getting too many vaccines.*
- *Overall, I am apprehensive face to vaccines.*
- *I consider vaccines to be effective in preventing disease.*
- *I think vaccines may weaken my immune system.*
- *In general, people around me are in favor of vaccination.*

The following table summarizes descriptive statistics about the above-described scores:

| **Score** | **Mean** | **SD** | **Range** | **Cronbach’s** $\boldsymbol{\alpha}$ |
| --- | --- | --- | --- | --- |
| Sense-of-Coherence 3-item (SOC-3) | 3.97 | 1.32 | (0-6) | 0.460 |
| Fear-of-COVID19 Scale (FCV-19S) | 16.86 | 6.31 | (7-35) | 0.901 |
| COVID-19 Risk Beliefs Score | 5.45 | 2.21 | (0-9) | 0.613 |
| Vaccine Trust Score | 5.16 | 1.47 | (0-6) | 0.808 |
| Vaccine Hesitancy Score | 9.21 | 6.27 | (0-32) | 0.794 |

# **Supplementary File 2** – Illustration of the choice-based exercise data structure

| **ID** | **Choice Task** | **Alternative** | **Choice** | **ASC** | **Age** |
| --- | --- | --- | --- | --- | --- |
| 4 | 1 | 1 | 1 | 1 | 59 |
| 4 | 1 | 2 | 0 | 1 | 59 |
| 4 | 1 | 3 | 0 | 0 | 59 |
| 4 | 2 | 1 | 0 | 1 | 59 |
| 4 | 2 | 2 | 1 | 1 | 59 |
| 4 | 2 | 3 | 0 | 0 | 59 |
| 4 | 3 | 1 | 0 | 1 | 59 |
| 4 | 3 | 2 | 1 | 1 | 59 |
| 4 | 3 | 3 | 0 | 0 | 59 |
| 4 | 4 | 1 | 0 | 1 | 59 |
| 4 | 4 | 2 | 1 | 1 | 59 |
| 4 | 4 | 3 | 0 | 0 | 59 |
| 4 | 5 | 1 | 0 | 1 | 59 |
| 4 | 5 | 2 | 1 | 1 | 59 |
| 4 | 5 | 3 | 0 | 0 | 59 |
| 4 | 6 | 1 | 0 | 1 | 59 |
| 4 | 6 | 2 | 1 | 1 | 59 |
| 4 | 6 | 3 | 0 | 0 | 59 |
| 4 | 7 | 1 | 1 | 1 | 59 |
| 4 | 7 | 2 | 0 | 1 | 59 |
| 4 | 7 | 3 | 0 | 0 | 59 |
| 4 | 8 | 1 | 1 | 1 | 59 |
| 4 | 8 | 2 | 0 | 1 | 59 |
| 4 | 8 | 3 | 0 | 0 | 59 |
| 4 | 9 | 1 | 1 | 1 | 59 |
| 4 | 9 | 2 | 0 | 1 | 59 |
| 4 | 9 | 3 | 0 | 0 | 59 |
| 4 | 10 | 1 | 0 | 1 | 59 |
| 4 | 10 | 2 | 1 | 1 | 59 |
| 4 | 10 | 3 | 0 | 0 | 59 |
| **4** | **11** | **1** | **0** | **1** | **59** |
| **4** | **11** | **2** | **0** | **1** | **59** |
| **4** | **11** | **3** | **1** | **0** | **59** |
| 4 | 12 | 1 | 1 | 1 | 59 |
| 4 | 12 | 2 | 0 | 1 | 59 |
| 4 | 12 | 3 | 0 | 0 | 59 |

Notes: ID, Choice task, Alternative, Choice, Alternative Specific Constant.

# **Supplementary File 3** – Details of excluded individuals

Before conducting analysis, we defined a list of exclusion criteria:

- Respondent chose vaccine optout at most 9 times over 12 in less than 1 minute.
- Respondent always chose option A or always chose option B.
- Respondent declared a poor or very poor quality of answers.
- Respondent declared not having answered to his best.

Over the 2037 observations included, 1883 (92%) were retained and 154 (8%) were dropped from the analysis. However, excluded individuals are slightly different from the main sample as shown in the tables below.

We performed a supplementary Mixed Logit considering the whole sample (n=2037) as sensibility analysis. Results did not differ (see Supplementary File 5).

Bivariate analysis indicates that the excluded respondents exhibited distinct characteristics, including a higher proportion of males, a younger age demographic, individuals with a single marital status, smokers, those employed in various occupational roles, and individuals with a comparatively higher level of education. These respondents also reported a lower perceived health status, displayed a greater inclination toward risk, and had a lower sense of coherence. Notably, they reported more pronounced impacts from the COVID-19 pandemic, as evidenced by higher levels of financial loss, a higher representation of essential workers, and a greater prevalence of having been afflicted by COVID-19 themselves. Additionally, their expressed fear of COVID-19 was more pronounced, while their perception of the associated risk was comparatively lower. In terms of vaccination, this group exhibited a lower rate of prior COVID-19 vaccination, and those who were vaccinated reported experiencing a higher frequency of side-effects. Importantly, these respondents displayed lower scores for vaccine trust and higher levels of vaccine hesitancy, signifying a notable contrast in their attitudes towards vaccination.

| **Sociodemographic characteristics** | **Non excluded respondents (n=1,883)** | **Excluded respondents (n=154)** | **Total (n=2,037)** | **P-value**^1^ |
| --- | --- | --- | --- | --- |
|  |  |  |  |  |
|  |  |  |  |  |
| **Gender** |  |  |  |  |
| Male | 48.86% | 59.09% | 49.63% | 0.001 |
| Female | 51.09% | 39.61% | 50.22% |  |
| Other | 0.05% | 1.30% | 0.15% |  |
|  |  |  |  |  |
| Male/Female Ratio | 0.96 | 1.49 | 0.99 | - |
| **Age (years)** |  |  |  |  |
| Mean | 51.32 | 37.84 | 50.30 | <0.001 |
| Standard deviation | 18.28 | 16.10 | 18.47 | 0.039 |
| Range | (18-94) | (18-92) | (18-94) |  |
|  |  |  |  |  |
| **Marital status** |  |  |  |  |
| Single | 29.79% | 49.35% | 31.27% | <0.001 |
| Married/living with a partner | 57.25% | 44.16% | 56.26% |  |
| Divorced/separated | 8.92% | 4.55% | 8.59% |  |
| Widowed | 4.04% | 1.95% | 3.88% |  |
| **Occupational status** |  |  |  |  |
| Employed | 46.68% | 57.14% | 47.47% | <0.001 |
| Retired | 36.91% | 15.58% | 35.30% |  |
| At home | 4.41% | 6.49% | 4.57% |  |
| Student | 5.20% | 10.39% | 5.60% |  |
| Unemployed | 4.62% | 7.14% | 4.81% |  |
| Sick and parental leave | 2.12% | 3.25% | 2.21% |  |
| Other | 0.05% | 0.00% | 0.05% |  |
| **Educational level** |  |  |  |  |
| Secondary or less and Diploma of professional studies | 35.85% | 35.71% | 35.84% | 0.437 |
| College and CEGEP | 28.20% | 23.38% | 27.84% |  |
| Baccalaureate, Master, and PhD | 35.85% | 40.91% | 36.23% |  |
| Other | 0.11% | 0.00% | 0.10% |  |
| **Annual household income (CAD)** |  |  |  |  |
| Mean | 67,752.26 | 58,587.66 | 67,059.40 | 0.010 |
| Standard deviation | 42,312.08 | 43,342.69 | 42,449.25 | 0.684 |
| Range | (2,500-165,000) | (2,500-165,000) | (2,500-165,000) |  |
| **Smoking** |  |  |  |  |
| Yes | 19.17% | 28.57% | 19.88% | 0.007 |
| No | 80.83% | 71.43% | 80.12% |  |
| **Living with an adult** |  |  |  |  |
| Yes | 72.28% | 63.64% | 71.62% | 0.028 |
| No | 27.72% | 36.36% | 28.38% |  |
| **Under-age dependent children (at least one)** |  |  |  |  |
| Yes | 20.02% | 35.71% | 21.21% | <0.001 |
| No | 79.98% | 64.29% | 78.79% |  |
| **Type of residence** |  |  |  |  |
| Rural | 27.83% | 26.62% | 27.74% | 0.820 |
| Urban | 72.17% | 73.38% | 72.26% |  |
| **Owning a home** |  |  |  |  |
| Yes | 63.62% | 55.19% | 62.98% | 0.046 |
| No | 36.38% | 44.81% | 37.02% |  |
| **Access to the outside** |  |  |  |  |
| Balcony | 53.90% | 56.49% | 54.10% | 0.592 |
| Terrace | 33.56% | 35.06% | 33.68% | 0.772 |
| Yard or garden | 66.33% | 43.51% | 64.60% | <0.001 |
| No access | 5.58% | 8.44% | 5.79% | 0.199 |
| **Live in the Grand Montréal** |  |  |  |  |
| Yes | 43.55% | 53.90% | 44.33% | 0.016 |
| No | 56.45% | 46.10% | 55.67% |  |
| **Health status** |  |  |  |  |
| Mean | 2.48 | 2.39 | 2.48 | 0.214 |
| Standard deviation | 0.89 | 0.93 | 0.89 | 0.462 |
| Range | (1-5) | (1-5) | (1-5) |  |
| **Satisfaction with health** |  |  |  |  |
| Mean | 7.06 | 6.88 | 7.05 | 0.287 |
| Standard deviation | 2.08 | 2.24 | 2.09 | 0.181 |
| Range | (0-10) | (0-10) | (0-10) |  |
| **Satisfaction with life** |  |  |  |  |
| Mean | 7.30 | 6.81 | 7.26 | 0.005 |
| Standard deviation | 2.06 | 2.42 | 2.09 | 0.004 |
| Range | (0-10) | (0-10) | (0-10) |  |
| **Willingness to take risks** |  |  |  |  |
| Mean | 4.68 | 5.67 | 4.76 | <0.001 |
| Standard deviation | 2.54 | 2.33 | 2.53 | 0.175 |
| Range | (0-10) | (0-10) | (0-10) |  |
| **Do you suffer from a disease or a physical or mental problem that reduces your quality of life (e.g., diabetes, cancer, osteoarthritis, etc.)?** | | | | |
| Yes | 28.25% | 19.48% | 27.59% | 0.025 |
| No | 71.75% | 80.52% | 72.41% |  |
| **SOC-3 Score (ranging from 0 to 6)**^2^ |  |  |  |  |
| Mean | 3.97 | 3.36 | 3.92 | <0.001 |
| Standard deviation | 1.32 | 1.18 | 1.32 | 0.066 |
| Range | (0-6) | (0-6) | (0-6) |  |
|  |  |  |  |  |

^1^The $p$-values refer to tests between groups using one-way analysis of variance, Kruskal-Wallis H test, Bartlett’s test for equality of variances, Fisher's exact test, and Chi2 test of independence.

^2^3-item Sense-of-Coherence questionnaire with 3-point Likert scale "No", "Yes, sometimes" and "Yes, usually" encoded 0, 1 and 2 for questions 1 and 2 and encoded 2, 1 and 0 for question 3. The score per individual is ranging from 0 to 6 and corresponds to the sum of each modality. See Supplementary File 1 for details.

| **COVID-19 experiences, COVID-19 vaccination, and vaccination perception** | **Non excluded respondents (n=1,883)** | **Excluded respondents (n=154)** | **Total (n=2,037)** | **P-value**^1^ |  |
| --- | --- | --- | --- | --- | --- |
|  |  |  |  |  |  |
|  |  |  |  |  |  |
| **Financial losses due to COVID-19** |  |  |  |  |  |
| No financial loss | 58.42% | 31.82% | 56.41% | <0.001 |  |
| Small financial losses | 30.54% | 39.61% | 31.22% |  |  |
| Fairly significant financial losses | 9.13% | 22.08% | 10.11% |  |  |
| Very significant financial losses | 1.91% | 6.49% | 2.26% |  |  |
| **Continued to work during the COVID-19 lockdown (from March to May 2020)** | | | |  |  |
| Yes | 45.25% | 66.88% | 46.88% | <0.001 |  |
| No | 54.75% | 33.12% | 53.12% |  |  |
| **Work status during the COVID-19 lockdown** |  |  |  |  |  |
| Essential worker (health worker) | 18.08% | 33.98% | 19.79% | <0.001 |  |
| Essential worker (other profession) | 50.59% | 50.49% | 50.58% |  |  |
| Non-essential worker | 31.34% | 15.53% | 29.63% |  |  |
| **Have you or a relative been sick with COVID-19 in the past few months?** | | |  |  |  |
| Yourself |  |  |  |  |  |
| Yes | 4.20% | 22.73% | 5.60% | <0.001 |  |
| No | 95.80% | 77.27% | 94.40% |  |  |
| A family member |  |  |  |  |  |
| Yes | 10.36% | 31.17% | 11.93% | <0.001 |  |
| No | 89.64% | 68.83% | 88.07% |  |  |
| A relative |  |  |  |  |  |
| Yes | 16.36% | 25.32% | 17.03% | 0.006 |  |
| No | 83.64% | 74.68% | 82.97% |  |  |
| **Fear of COVID-19 Score (ranging from 7 to 35)**^2^ | |  |  |  |  |
| Mean | 16.86 | 20.49 | 17.14 | <0.001 |  |
| Standard deviation | 6.31 | 6.83 | 6.42 | 0.177 |  |
| Range | (7-35) | (7-35) | (7-35) |  |  |
| **COVID-19 vaccination intention** |  |  |  |  |  |
| Already vaccinated | 43.23% | 33.77% | 42.51% | 0.013 |  |
| Not vaccinated but plan to be vaccinated | 45.35% | 48.05% | 45.56% |  |  |
| Not vaccinated and do not plan to be vaccinated | 11.42% | 18.18% | 11.93% |  |  |
| **Side effects following the injection** |  |  |  |  |  |
| No side effect | 67.20% | 57.69% | 66.63% | 0.209 |  |
| Pain or itching | 27.15% | 30.77% | 27.37% | 0.684 |  |
| Fever | 4.67% | 11.54% | 5.08% | 0.042 |  |
| Redness at the injection site | 5.04% | 9.62% | 5.31% | 0.189 |  |
| Have been hospitalized | 0.12% | 0.00% | 0.12% | 0.999 |  |
| **COVID-19 Risk Beliefs Score**^3^ |  |  |  |  |  |
| Mean | 5.45 | 3.85 | 5.32 | <0.001 |  |
| Standard deviation | 2.21 | 2.12 | 2.24 | 0.494 |  |
| Range | (0-9) | (0-9) | (0-9) |  |  |
| **Vaccine Trust Score (ranging from 0 to 6)**^4^ |  |  |  |  |  |
| Mean | 5.16 | 4.15 | 5.09 | <0.001 |  |
| Standard deviation | 1.47 | 1.97 | 1.54 | <0.001 |  |
| Range | (0-6) | (0-6) | (0-6) |  |  |
| **Vaccine Hesitancy Score (ranging from 0 to 32)**^5^ | |  |  |  |  |
| Mean | 9.21 | 13.76 | 9.55 | <0.001 |  |
| Standard deviation | 6.27 | 5.03 | 6.30 | 0.001 |  |
| Range | (0-32) | (0-32) | (0-32) |  |  |
|  |  |  |  |  |  |

^1^The $p$-values refer to tests between groups using one-way analysis of variance, Kruskal-Wallis H test, Bartlett’s test for equality of variances, Fisher's exact test, and Chi2 test of independence.

^2^The Fear of COVID-19 score is based on the 7-item Fear of COVID-19 Scale (FCV-19S) with 5-point Likert scale from "Strongly disagree" (1) to "Strongly agree" (5). The score is ranging from 7 to 35 and corresponds to the sum of each modality. See Supplementary File 1 for details.

^3^The COVID-19 Risk Beliefs Score corresponds to the sum of a 3-item set of questions about the perception of the pandemic and the vaccination. The 3 items were scored with a 4-point Likert scale ranging from "Strongly disagree" (0 or 3) to "Strongly agree" (3 or 0). The score ranged from 0 (lowest risk perception) to 9 (highest risk perception). See Supplementary File 1 for details.

^4^The vaccine trust score is based on four questions) and is ranging from 0 to 6. The "Yes/No" questions are encoded 1 and 0 and the fourth question is encoded 0, 1 and 2. The score corresponds to the sum of each modality. See Supplementary File 1 for details.

^5^The vaccine hesitancy score is based on eight questions with a 5-point Likert scale ranging from "Strongly disagree" (0 or 4) to "Strongly agree" (4 or 0). The modality "Do not know" is encoded 2 and questions 3, 4, 5 and 7 have been reversed. The score per individual corresponds to the sum of each modality. See Supplementary File 1 for details.

| **DCE Perception** | **Non excluded respondents (n=1,883)** | **Excluded respondents (n=154)** | **Total (n=2,037)** | **P-value**^1^ |  |
| --- | --- | --- | --- | --- | --- |
|  |  |  |  |  |  |
|  |  |  |  |  |  |
| **Response time to the DCE*** |  |  |  |  |  |
| Mean | 05:18 | 01:26 | 05:01 | <0.001 |  |
| Standard deviation | 05:57 | 01:13 | 05:50 | <0.001 |  |
| Median | 04:10 | 00:59 | 03:59 |  |  |
| Range | (00:36-01:22:06) | (00:33-09:04) | (00:33-01:22:06) |  |  |
| **Choice certainty score (ranging from 0 to 120)**^2^ | |  |  |  |  |
| Mean | 95.45 | 77.49 | 94.09 | <0.001 |  |
| Standard deviation | 20.66 | 25.41 | 21.57 | <0.001 |  |
| Range | (0-120) | (0-120) | (0-120) |  |  |
| **Choice exercise difficulty** |  |  |  |  |  |
| Very easy | 17.79% | 14.94% | 17.57% | 0.050 |  |
| Easy | 35.74% | 33.12% | 35.54% |  |  |
| Neutral | 41.69% | 42.86% | 41.78% |  |  |
| Hard | 4.62% | 7.79% | 4.86% |  |  |
| Very hard | 0.16% | 1.30% | 0.25% |  |  |
| **Number of dimensions considered** |  |  |  |  |  |
| 1 | 6.27% | 18.83% | 7.22% | <0.001 |  |
| 2 | 28.50% | 36.36% | 29.09% |  |  |
| 3 or more | 53.64% | 23.38% | 51.35% |  |  |
| Not sure | 11.59% | 21.43% | 12.33% |  |  |
| **Quality of responses** |  |  |  |  |  |
| Very good | 38.58% | 20.92% | 37.24% | <0.001 |  |
| Good | 46.70% | 40.52% | 46.23% |  |  |
| Average | 14.72% | 30.72% | 15.94% |  |  |
| Bad* | 0.00% | 5.23% | 0.40% |  |  |
| Very bad* | 0.00% | 2.61% | 0.20% |  |  |
| **Have been annoyed** |  |  |  |  |  |
| Yes | 19.18% | 41.83% | 20.90% | <0.001 |  |
| No | 80.82% | 58.17% | 79.10% |  |  |
| **Percentage of vaccine refusals** |  |  |  |  |  |
| Never chose to refuse the vaccine | 61.55% | 57.14% | 61.22% | 0.515 |  |
| Chose to refuse the vaccine 1 to 3 times | 19.70% | 20.13% | 19.73% |  |  |
| Chose to refuse the vaccine 4 to 7 times | 9.82% | 13.64% | 10.11% |  |  |
| Chose to refuse the vaccine 8 to 11 times | 4.04% | 5.19% | 4.12% |  |  |
| Always chose to refuse the vaccine* | 4.89% | 3.90% | 4.81% |  |  |
|  |  |  |  |  |  |
| Failed to the rationality test | 5.52% | 3.03% | 8.55% | <0.001 |  |
| Failed to the temporal consistency test | 24.16% | 3.13% | 27.30% | <0.001 |  |
|  |  |  |  |  |  |

Note: *refers to exclusion criteria.

^1^The $p$-values refer to tests between groups using one-way analysis of variance, Kruskal-Wallis H test, Bartlett’s test for equality of variances, Fisher's exact test, and Chi2 test of independence.

^2^The choice certainty score corresponds to the sum of each twelve certitude questions "Are you sure of your choice?" scaled between 0 to 10. The score is thus ranging from 0 to 120.

# **Supplementary File 4** – Multinomial mixed logit model sensitivity analyses

Four different specifications were run as sensitivity analyses.

- Among $2,037$ respondents who completed the DCE, we excluded a few because they responded to the twelve choice tasks in less than one minute and chose 75% of their time or less to refuse the vaccine ($n=90$), always chose the option A ($n=22$) or the option B ($n=6$), declared a poor ($n=52$) or very poor ($n=8$) quality of answers, or because they declared not having answered to their best ($n=4$). A total of $154$ respondents (i.e., $7.56\%$) were dropped from the analysis yielding a full sample of $1,883$. We performed the same specification as defined in the manuscript including those respondents. The conditional relative weight importance of the attributes was as follows: vaccine effectiveness ($32.91\%$), side effects of the vaccine ($24.32\%$), results’ publication ($22.15\%$), number of shots to be received ($15.15\%$), priority population ($4.06\%$), type of vaccine ($0.76\%$), and vaccination location ($0.66\%$).
- In total, $104/1883$ respondents failed the rationality test (i.e., chose the vaccine alternative B which was supposed to be “dominated” by the vaccine alternative A) and $455/1883$ respondents failed the temporal consistency test (i.e., did not chose the same vaccine alternative at choice tasks 2 and 12). To note that we did not consider respondents who chose the opt-out alternative at the first choice task as respondents who failed the rationality test ($n=139$). The conditional relative weight importance of the attributes was as follows: vaccine effectiveness ($32.78\%$), side effects of the vaccine ($25.07\%$), results’ publication ($22.42\%$), number of shots to be received ($15.78\%$), type of vaccine ($1.76\%$), vaccination location ($1.15\%$), and priority population ($1.05\%$).
- We asked respondents whether they were already vaccinated or if they intended to get vaccinated or not (“What is your intention to get vaccinated against COVID-19?”). Being already vaccinated could create a bias in the results (e.g., because vaccination was not important anymore for them). Thus, we performed the MXL model for vaccinated respondents only. The conditional relative weight importance of the attributes was as follows: vaccine effectiveness ($35.40\%$), side effects of the vaccine ($23.96\%$), results’ publication ($22.94\%$), number of shots to be received ($11.81\%$), priority population ($4.88\%$), vaccination location ($0.73\%$), and type of vaccine ($0.29\%$).
- In total, $87/1,883$ respondents ($4.62\%$) and $3/1,883$ ($0.16\%$) found the discrete choice exercise as being “hard” or “very hard”, respectively. We performed the same specification as defined in the manuscript excluding those respondents ($n=1,793$). The conditional relative weight importance of the attributes was as follows: vaccine effectiveness ($32.81\%$), side effects of the vaccine ($25.41\%$), results’ publication ($22.14\%$), number of shots to be received ($15.23\%$), priority population ($3.55\%$), type of vaccine ($0.73\%$), and vaccination location ($0.13\%$).

| **Attribute** | **Mixed logit model** | | | | | | | | | | | | | | | | | | | |
| --- | --- | --- | --- | --- | --- | --- | --- | --- | --- | --- | --- | --- | --- | --- | --- | --- | --- | --- | --- | --- |
|  | **Retained sample** | | | | **With respondents excluded from the retained sample** | | | | **Without respondents who declared not being vaccinated against COVID-19** | | | | **Without respondents who declared the DCE being "hard" or "very hard"** | | | | **Without respondents who failed to the rationality test or to the temporal consistency test** | | | |
|  | **Mean** | | **Standard deviation** | | **Mean** | | **Standard deviation** | | **Mean** | | **Standard deviation** | | **Mean** | | **Standard deviation** | | **Mean** | | **Standard deviation** | |
|  | **Coefficient** | **P-value** | **Coefficient** | **P-value** | **Coefficient** | **P-value** | **Coefficient** | **P-value** | **Coefficient** | **P-value** | **Coefficient** | **P-value** | **Coefficient** | **P-value** | **Coefficient** | **P-value** | **Coefficient** | **P-value** | **Coefficient** | **P-value** |
|  |  |  |  |  |  |  |  |  |  |  |  |  |  |  |  |  |  |  |  |  |
| **ASC** | 6.294*** | <.001 | 4.332*** | <.001 | 5.457*** | <.001 | 4.150*** | <.001 | 6.517*** | <.001 | 3.162*** | <.001 | 6.073*** | <.001 | 4.238*** | <.001 | 7.673*** | <.001 | 5.427*** | <.001 |
|  |  |  |  |  |  |  |  |  |  |  |  |  |  |  |  |  |  |  |  |  |
| **Type of vaccine** |  |  |  |  |  |  |  |  |  |  |  |  |  |  |  |  |  |  |  |  |
| Viral vector vaccine | (reference) |  | (reference) |  |  |  |  |  | (reference) |  | (reference) |  | (reference) |  | (reference) |  | (reference) |  | (reference) |  |
| mRNA vaccine | 0.035 | 0.528 | 0.393** | 0.002 | 0.006 | 0.902 | 0.084 | 0.613 | 0.098 | 0.188 | 0.088 | 0.746 | 0.008 | 0.885 | 0.105 | 0.642 | -0.054 | 0.468 | -0.541*** | <.001 |
| Subunit vaccine | -0.082 | 0.148 | 0.442*** | <.001 | -0.075 | 0.122 | -0.359** | 0.005 | -0.014 | 0.862 | 0.486*** | <.001 | -0.072 | 0.182 | 0.307* | 0.011 | -0.273*** | <.001 | 0.554*** | <.001 |
| DNA vaccine | -0.094 | 0.088 | 0.224 | 0.172 | -0.095* | 0.045 | 0.221* | 0.044 | -0.054 | 0.469 | 0.060 | 0.705 | -0.088 | 0.099 | 0.098 | 0.495 | -0.201** | 0.006 | 0.169 | 0.520 |
| Inactivated vaccine | 0.038 | 0.492 | 0.597*** | <.001 | 0.027 | 0.569 | 0.390*** | <.001 | -0.002 | 0.975 | -0.591*** | <.001 | 0.022 | 0.670 | 0.430*** | <.001 | 0.074 | 0.322 | -0.846*** | <.001 |
| Live-attenuated vaccine | -0.116* | 0.039 | 0.398** | 0.001 | -0.089 | 0.064 | 0.333** | 0.005 | -0.097 | 0.207 | -0.360* | 0.014 | -0.116* | 0.031 | -0.380*** | <.001 | -0.350*** | <.001 | -0.579*** | <.001 |
| **Vaccine effectiveness** |  |  |  |  |  |  |  |  |  |  |  |  |  |  |  |  |  |  |  |  |
| 95% | (reference) |  | (reference) |  | (reference) |  | (reference) |  | (reference) |  | (reference) |  | (reference) |  | (reference) |  | (reference) |  | (reference) |  |
| 85% | -0.656*** | <.001 | 0.153 | 0.239 | -0.549*** | <.001 | -0.085 | 0.323 | -0.637*** | <.001 | 0.111 | 0.399 | -0.614*** | <.001 | -0.063 | 0.454 | -0.795*** | <.001 | -0.208 | 0.204 |
| 75% | -1.380*** | <.001 | 0.393** | 0.001 | -1.146*** | <.001 | -0.220 | 0.105 | -1.285*** | <.001 | 0.358** | 0.009 | -1.315*** | <.001 | -0.328** | 0.001 | -1.806*** | <.001 | 0.491*** | <.001 |
| 60% | -2.563*** | <.001 | 1.379*** | <.001 | -2.124*** | <.001 | 1.215*** | <.001 | -2.374*** | <.001 | 1.192*** | <.001 | -2.443*** | <.001 | 1.279*** | <.001 | -3.286*** | <.001 | 1.532*** | <.001 |
| **Side effects of the vaccine** |  |  |  |  |  |  |  |  |  |  |  |  |  |  |  |  |  |  |  |  |
| None | (reference) |  | (reference) |  | (reference) |  | (reference) |  | (reference) |  | (reference) |  | (reference) |  | (reference) |  | (reference) |  | (reference) |  |
| 1 in 1000 chance of having a minor effect | -0.536*** | <.001 | 0.369*** | <.001 | -0.425*** | <.001 | 0.181* | 0.042 | -0.472*** | <.001 | 0.188 | 0.186 | -0.505*** | <.001 | -0.351*** | <.001 | -0.680*** | <.001 | -0.311** | 0.007 |
| 1 in 10000 chances of having a major effect | -1.414*** | <.001 | 1.411*** | <.001 | -1.147*** | <.001 | 1.162*** | <.001 | -1.143*** | <.001 | 1.064*** | <.001 | -1.381*** | <.001 | 1.264*** | <.001 | -1.837*** | <.001 | 1.717*** | <.001 |
| **Vaccination location** |  |  |  |  |  |  |  |  |  |  |  |  |  |  |  |  |  |  |  |  |
| A drugstore | (reference) |  |  |  | (reference) |  | (reference) |  | (reference) |  | (reference) |  | (reference) |  | (reference) |  | (reference) |  | (reference) |  |
| A care center | 0.003 | 0.936 | 0.170 | 0.327 | -0.005 | 0.885 | -0.288*** | <.001 | 0.023 | 0.643 | 0.343*** | <.001 | 0.017 | 0.631 | 0.093 | 0.340 | 0.066 | 0.184 | 0.361* | 0.013 |
| A sports center, an exhibition center | -0.023 | 0.532 | 0.306** | 0.002 | -0.034 | 0.277 | -0.160 | 0.137 | 0.021 | 0.673 | -0.166 | 0.214 | -0.008 | 0.813 | 0.242** | 0.009 | 0.038 | 0.440 | 0.458*** | <.001 |
| **Priority population to receive the vaccine** |  |  |  |  |  |  |  |  |  |  |  |  |  |  |  |  |  |  |  |  |
| No priority population | (reference) |  | (reference) |  | (reference) |  | (reference) |  | (reference) |  | (reference) |  | (reference) |  | (reference) |  | (reference) |  | (reference) |  |
| Vulnerable people with a great loss of autonomy | -0.034 | 0.350 | 0.268* | 0.025 | -0.045 | 0.154 | -0.308*** | <.001 | -0.039 | 0.446 | 0.360*** | <.001 | -0.037 | 0.298 | 0.243** | 0.002 | 0.057 | 0.256 | -0.429*** | <.001 |
| Primary and secondary teachers | -0.251*** | <.001 | 0.397*** | <.001 | -0.216*** | <.001 | 0.175 | 0.090 | -0.291*** | <.001 | -0.176 | 0.230 | -0.226*** | <.001 | -0.175* | 0.048 | -0.171** | 0.001 | 0.282* | 0.032 |
| **Number of shots to be received** |  |  |  |  |  |  |  |  |  |  |  |  |  |  |  |  |  |  |  |  |
| 1 shot | (reference) |  | (reference) |  | (reference) |  | (reference) |  | (reference) |  | (reference) |  | (reference) |  | (reference) |  | (reference) |  | (reference) |  |
| 2 shots | -0.228*** | <.001 | 0.352*** | <.001 | -0.184*** | <.001 | -0.165 | 0.209 | -0.014 | 0.775 | 0.278** | 0.008 | -0.217*** | <.001 | -0.418*** | <.001 | -0.311*** | <.001 | -0.442** | 0.003 |
| 3 shots | -0.928*** | <.001 | 0.948*** | <.001 | -0.728*** | <.001 | 0.700*** | <.001 | -0.717*** | <.001 | 0.635*** | <.001 | -0.838*** | <.001 | 0.751*** | <.001 | -1.168*** | <.001 | 0.989*** | <.001 |
| **Vaccine whose results have been published in a scientific journal** |  |  |  |  |  |  |  |  |  |  |  |  |  |  |  |  |  |  |  |  |
| Results published in an international journal | (reference) |  | (reference) |  | (reference) |  | (reference) |  | (reference) |  | (reference) |  | (reference) |  | (reference) |  | (reference) |  | (reference) |  |
| Results published in a national journal | -0.148*** | <.001 | 0.037 | 0.732 | -0.146*** | <.001 | -0.075 | 0.187 | -0.149** | 0.001 | 0.071 | 0.468 | -0.151*** | <.001 | -0.118 | 0.165 | -0.210*** | <.001 | -0.069 | 0.473 |
| Unpublished results | -1.461*** | <.001 | 1.325*** | <.001 | -1.156*** | <.001 | 1.109*** | <.001 | -1.259*** | <.001 | 1.176*** | <.001 | -1.340*** | <.001 | 1.149*** | <.001 | -1.823*** | <.001 | 1.613*** | <.001 |
|  |  |  |  |  |  |  |  |  |  |  |  |  |  |  |  |  |  |  |  |  |
| **Relative importance weights of attributes** |  |  |  |  |  |  |  |  |  |  |  |  |  |  |  |  |  |  |  |  |
| Type of vaccine | 0.61% |  |  |  | 0.76% |  |  |  | 0.29% |  |  |  | 0.73% |  |  |  | 1.76% |  |  |  |
| Vaccine effectiveness | 32.50% |  |  |  | 32.91% |  |  |  | 35.40% |  |  |  | 32.81% |  |  |  | 32.78% |  |  |  |
| Side effects of the vaccine | 24.76% |  |  |  | 24.32% |  |  |  | 23.96% |  |  |  | 25.41% |  |  |  | 25.07% |  |  |  |
| Vaccination location | 0.28% |  |  |  | 0.66% |  |  |  | 0.73% |  |  |  | 0.13% |  |  |  | 1.15% |  |  |  |
| Priority population to receive the vaccine | 3.60% |  |  |  | 4.06% |  |  |  | 4.88% |  |  |  | 3.55% |  |  |  | 1.05% |  |  |  |
| Number of shots to be received | 15.73% |  |  |  | 15.15% |  |  |  | 11.81% |  |  |  | 15.23% |  |  |  | 15.78% |  |  |  |
| Vaccine whose results have been published in a scientific journal | 22.51% |  |  |  | 22.15% |  |  |  | 22.94% |  |  |  | 22.14% |  |  |  | 22.42% |  |  |  |
|  |  |  |  |  |  |  |  |  |  |  |  |  |  |  |  |  |  |  |  |  |
| Observations | 67,788 |  |  |  | 73,332 |  |  |  | 29,304 |  |  |  | 64,548 |  |  |  | 49,032 |  |  |  |
| Chi2 | 9531.71 |  |  |  | 9910.42 |  |  |  | 2158.42 |  |  |  | 9002.55 |  |  |  | 8639.21 |  |  |  |
| Log likelihood | -14,522.79 |  |  |  | -16,484.99 |  |  |  | -6227.56 |  |  |  | -13,781.95 |  |  |  | -9055.33 |  |  |  |
| Null log likelihood | -19,288.64 |  |  |  | -21,440.20 |  |  |  | -7306.77 |  |  |  | -18,283.22 |  |  |  | -13374.94 |  |  |  |
| AIC | 29,121.58 |  |  |  | 33,045.99 |  |  |  | 12,531.12 |  |  |  | 27,639.89 |  |  |  | 18186.66 |  |  |  |
| BIC | 29,256.94 |  |  |  | 33,395.69 |  |  |  | 12,845.97 |  |  |  | 27,984.75 |  |  |  | 18521.07 |  |  |  |

ASC: Alternative Specific Constant; AIC: Akaike Information Criterion; BIC: Bayesian Information Criterion; *p<0.05, ** p<0.01, *** p<0.001. Note: 200 Halton draws specified.

# **Supplementary File 5** - Respondent’s personal ranking of attributes

# **Supplementary File 6** – Details of “anti-vaccine” individuals (refused 100% of the times vaccines alternatives)

| **Sociodemographic characteristics** | **Chose to accept the vaccine at least once (n=1,791)** | **Always chose to refuse the vaccine (n=92)** | **Total (n=1,883)** | **P-value**^1^ |  |
| --- | --- | --- | --- | --- | --- |
|  |  |  |  |  |  |
|  |  |  |  |  |  |
| **Gender** |  |  |  |  |  |
| Male | 48.97% | 46.74% | 48.86% | 0.761 |  |
| Female | 50.98% | 53.26% | 51.09% |  |  |
| Other | 0.06% | 0.00% | 0.05% |  |  |
|  |  |  |  |  |  |
| Male/Female Ratio | 0.96 | 0.88 | 0.96 |  |  |
| **Age (years)** |  |  |  |  |  |
| Mean | 51.52 | 47.40 | 51.32 | 0.035 |  |
| Standard deviation | 18.39 | 15.52 | 18.28 | 0.034 |  |
| Range | (18-94) | (23-81) | (18-94) |  |  |
|  |  |  |  |  |  |
| **Marital status** |  |  |  |  |  |
| Single | 29.59% | 33.70% | 29.79% | 0.815 |  |
| Married/living with a partner | 57.40% | 54.35% | 57.25% |  |  |
| Divorced/separated | 8.99% | 7.61% | 8.92% |  |  |
| Widowed | 4.02% | 4.35% | 4.04% |  |  |
| **Occupational status** |  |  |  |  |  |
| Employed | 46.51% | 50.00% | 46.68% | <0.001 |  |
| Retired | 37.74% | 20.65% | 36.91% |  |  |
| At home | 4.13% | 9.78% | 4.41% |  |  |
| Student | 5.42% | 1.09% | 5.20% |  |  |
| Unemployed | 4.19% | 13.04% | 4.62% |  |  |
| Sick and parental leave | 1.95% | 5.43% | 2.12% |  |  |
| Other | 0.06% | 0.00% | 0.05% |  |  |
| **Educational level** |  |  |  |  |  |
| Secondary or less and Diploma of professional studies | 34.84% | 55.43% | 35.85% | 0.001 |  |
| College and CEGEP | 28.48% | 22.83% | 28.20% |  |  |
| Baccalaureate, Master and PhD | 36.57% | 21.74% | 35.85% |  |  |
| Other | 0.11% | 0.00% | 0.11% |  |  |
| **Annual household income (CAD)** |  |  |  |  |  |
| Mean | 68,263.54 | 57,798.91 | 67,752.26 | 0.021 |  |
| Standard deviation | 42,338.34 | 40,754.97 | 42,312.08 | 0.621 |  |
| Range | (2,500-165,000) | (7,500-165,000) | (2,500-165,000) |  |  |
| **Smoking** |  |  |  |  |  |
| Yes | 18.76% | 27.17% | 19.17% | 0.062 |  |
| No | 81.24% | 72.83% | 80.83% |  |  |
| **Living with an adult** |  |  |  |  |  |
| Yes | 72.36% | 70.65% | 72.28% | 0.812 |  |
| No | 27.64% | 29.35% | 27.72% |  |  |
| **Under-age dependent children (at least one)** |  |  |  |  |  |
| Yes | 19.71% | 26.09% | 20.02% | 0.175 |  |
| No | 80.29% | 73.91% | 79.98% |  |  |
| **Type of residence** |  |  |  |  |  |
| Rural | 27.41% | 35.87% | 27.83% | 0.100 |  |
| Urban | 72.59% | 64.13% | 72.17% |  |  |
| **Owning a home** |  |  |  |  |  |
| Yes | 64.15% | 53.26% | 63.62% | 0.045 |  |
| No | 35.85% | 46.74% | 36.38% |  |  |
| **Access to the outside** |  |  |  |  |  |
| Balcony | 53.10% | 69.57% | 53.90% | 0.002 |  |
| Terrace | 33.28% | 39.13% | 33.56% | 0.246 |  |
| Yard or garden | 66.16% | 69.57% | 66.33% | 0.501 |  |
| No access | 5.75% | 2.17% | 5.58% | 0.145 |  |
| **Live in the Grand Montréal** |  |  |  |  |  |
| Yes | 43.66% | 41.30% | 43.55% | 0.656 |  |
| No | 56.34% | 58.70% | 56.45% |  |  |
| **Health status** |  |  |  |  |  |
| Mean | 2.48 | 2.55 | 2.48 | 0.430 |  |
| Standard deviation | 0.88 | 1.04 | 0.89 | 0.023 |  |
| Range | (1-5) | (1-5) | (1-5) |  |  |
| **Satisfaction with health** |  |  |  |  |  |
| Mean | 7.07 | 6.96 | 7.06 | 0.613 |  |
| Standard deviation | 2.06 | 2.35 | 2.08 | 0.070 |  |
| Range | (0-10) | (0-10) | (0-10) |  |  |
| **Satisfaction with life** |  |  |  |  |  |
| Mean | 7.33 | 6.76 | 7.30 | 0.010 |  |
| Standard deviation | 2.02 | 2.67 | 2.06 | <0.001 |  |
| Range | (0-10) | (0-10) | (0-10) |  |  |
| **Willingness to take risks** |  |  |  |  |  |
| Mean | 4.69 | 4.54 | 4.68 | 0.606 |  |
| Standard deviation | 2.52 | 2.90 | 2.54 | 0.052 |  |
| Range | (0-10) | (0-10) | (0-10) |  |  |
| **Do you suffer from a disease or a physical or mental problem that reduces your quality of life (e.g., diabetes, cancer, osteoarthritis)?** | | | | |  |
| Yes | 28.48% | 23.91% | 28.25% | 0.343 |  |
| No | 71.52% | 76.09% | 71.75% |  |  |
| **SOC-3 Score (ranging from 0 to 6)**^2^ |  |  |  |  |  |
| Mean | 3.98 | 3.75 | 3.97 | 0.104 |  |
| Standard deviation | 1.31 | 1.43 | 1.32 | 0.247 |  |
| Range | (0-6) | (0-6) | (0-6) |  |  |
|  |  |  |  |  |  |

^1^The $p$-values refer to tests between groups using one-way analysis of variance, Kruskal-Wallis H test, Bartlett’s test for equality of variances, Fisher's exact test, and Chi2 test of independence.

^2^3-item Sense-of-Coherence questionnaire with 3-point Likert scale "No", "Yes, sometimes" and "Yes, usually" encoded 0, 1 and 2 for questions 1 and 2 and encoded 2, 1 and 0 for question 3. The score per individual is ranging from 0 to 6 and corresponds to the sum of each modality. See Supplementary File 1 for details.

| **COVID-19 experiences, COVID-19 vaccination, and vaccination perception** | **Chose to accept the vaccine at least once (n=1,791)** | **Always chose to refuse the vaccine (n=92)** | **Total (n=1,883)** | **P-value**^1^ |  |
| --- | --- | --- | --- | --- | --- |
|  |  |  |  |  |  |
|  |  |  |  |  |  |
| **Financial losses due to COVID-19** |  |  |  |  |  |
| No financial loss | 58.57% | 55.43% | 58.42% | 0.211 |  |
| Small financial losses | 30.65% | 28.26% | 30.54% |  |  |
| Fairly significant financial losses | 8.99% | 11.96% | 9.13% |  |  |
| Very significant financial losses | 1.79% | 4.35% | 1.91% |  |  |
| **Continued to work during the COVID-19 lockdown (from March to May 2020)** | | |  |  |  |
| Yes | 45.51% | 40.22% | 45.25% | 0.320 |  |
| No | 54.49% | 59.78% | 54.75% |  |  |
| **Work status during the COVID-19 lockdown** |  |  |  |  |  |
| Essential worker (health worker) | 18.40% | 10.81% | 18.08% | 0.304 |  |
| Essential worker (other profession) | 50.06% | 62.16% | 50.59% |  |  |
| Non-essential worker | 31.53% | 27.03% | 31.34% |  |  |
| **Have you or a relative been sick with COVID-19 in the past few months?** | | |  |  |  |
| Yourself |  |  |  |  |  |
| Yes | 4.19% | 4.35% | 4.20% | 0.793 |  |
| No | 95.81% | 95.65% | 95.80% |  |  |
| A family member |  |  |  |  |  |
| Yes | 10.61% | 5.43% | 10.36% | 0.112 |  |
| No | 89.39% | 94.57% | 89.64% |  |  |
| A relative |  |  |  |  |  |
| Yes | 16.58% | 11.96% | 16.36% | 0.242 |  |
| No | 83.42% | 88.04% | 83.64% |  |  |
| **Fear of COVID-19 Score (ranging from 7 to 35)**^2^ |  |  |  |  |  |
| Mean | 17.07 | 12.93 | 16.86 | <0.001 |  |
| Standard deviation | 6.21 | 7.00 | 6.31 | 0.103 |  |
| Range | (7-35) | (7-35) | (7-35) |  |  |
| **COVID-19 vaccination intention** |  |  |  |  |  |
| Already vaccinated | 45.23% | 4.35% | 43.23% | <0.001 |  |
| Not vaccinated but plan to be vaccinated | 47.29% | 7.61% | 45.35% |  |  |
| Not vaccinated and do not plan to be vaccinated | 7.48% | 88.04% | 11.42% |  |  |
| **Side effects following the injection** |  |  |  |  |  |
| No side effect | 67.04% | 100.00% | 67.20% | 0.309 |  |
| Pain or itching | 27.28% | 0.00% | 27.15% | 0.579 |  |
| Fever | 4.69% | 0.00% | 4.67% | 0.999 |  |
| Redness at the injection site | 5.06% | 0.00% | 5.04% | 0.999 |  |
| Have been hospitalized | 0.12% | 0.00% | 0.12% | 0.999 |  |
| **COVID-19 Risk Beliefs Score**^3^ |  |  |  |  |  |
| Mean | 5.60 | 2.49 | 5.45 | <0.001 |  |
| Standard deviation | 2.11 | 1.95 | 2.21 | 0.349 |  |
| Range | (0-9) | (0-7) | (0-9) |  |  |
| **Vaccine Trust Score (ranging from 0 to 6)**^4^ |  |  |  |  |  |
| Mean | 5.35 | 1.58 | 5.16 | <0.001 |  |
| Standard deviation | 1.19 | 1.90 | 1.47 | <0.001 |  |
| Range | (0-6) | (0-6) | (0-6) |  |  |
| **Vaccine Hesitancy Score (ranging from 0 to 32)**^5^ |  |  |  |  |  |
| Mean | 8.72 | 18.69 | 9.21 | <0.001 |  |
| Standard deviation | 5.89 | 5.97 | 6.27 | 0.864 |  |
| Range | (0-31) | (4-32) | (0-32) |  |  |
|  |  |  |  |  |  |

^1^The $p$-values refer to tests between groups using one-way analysis of variance, Kruskal-Wallis H test, Bartlett’s test for equality of variances, Fisher's exact test, and Chi2 test of independence.

^2^The Fear of COVID-19 score is based on the 7-item Fear of COVID-19 Scale (FCV-19S) with 5-point Likert scale from "Strongly disagree" (1) to "Strongly agree" (5). The score is ranging from 7 to 35 and corresponds to the sum of each modality. See Supplementary File 1 for details.

^3^The COVID-19 Risk Beliefs Score corresponds to the sum of a 3-item set of questions about the perception of the pandemic and the vaccination. The 3 items were scored with a 4-point Likert scale ranging from "Strongly disagree" (0 or 3) to "Strongly agree" (3 or 0). The score ranged from 0 (lowest risk perception) to 9 (highest risk perception). See Supplementary File 1 for details.

^4^The vaccine trust score is based on four questions) and is ranging from 0 to 6. The "Yes/No" questions are encoded 1 and 0 and the fourth question is encoded 0, 1 and 2. The score corresponds to the sum of each modality. See Supplementary File 1 for details.

^5^The vaccine hesitancy score is based on eight questions with a 5-point Likert scale ranging from "Strongly disagree" (0 or 4) to "Strongly agree" (4 or 0). The modality "Do not know" is encoded 2 and questions 3, 4, 5 and 7 have been reversed. The score per individual corresponds to the sum of each modality. See Supplementary File 1 for details.

| **DCE Perception** | **Chose to accept the vaccine at least once (n=1,791)** | **Always chose to refuse the vaccine (n=92)** | **Total (n=1,883)** | **P-value**^1^ |  |
| --- | --- | --- | --- | --- | --- |
|  |  |  |  |  |  |
|  |  |  |  |  |  |
| **Response time to the DCE** |  |  |  |  |  |
| Mean | 05:25 | 03:07 | 05:18 | <0.001 |  |
| Standard deviation | 05:52 | 07:13 | 05:57 | 0.003 |  |
| Median | 04:19 | 01:41 | 04:10 |  |  |
| Range | (00:51-01:22:06) | (00:36-01:08:01) | (00:36-01:22:06) |  |  |
| **Choice certainty score (ranging from 0 to 120)**^2^ |  |  |  |  |  |
| Mean | 94.98 | 104.66 | 95.45 | <0.001 |  |
| Standard deviation | 20.03 | 29.04 | 20.66 | <0.001 |  |
| Range | (0-120) | (0-120) | (0-120) |  |  |
| **Choice exercise difficulty** |  |  |  |  |  |
| Very easy | 15.91% | 54.35% | 17.79% | <0.001 |  |
| Easy | 36.96% | 11.96% | 35.74% |  |  |
| Neutral | 42.27% | 30.43% | 41.69% |  |  |
| Hard | 4.69% | 3.26% | 4.62% |  |  |
| Very hard | 0.17% | 0.00% | 0.16% |  |  |
| **Number of dimensions considered** |  |  |  |  |  |
| 1 | 5.37% | 23.91% | 6.27% | <0.001 |  |
| 2 | 29.51% | 8.70% | 28.50% |  |  |
| 3 or more | 55.34% | 20.65% | 53.64% |  |  |
| Not sure | 9.78% | 46.74% | 11.59% |  |  |
| **Quality of responses** |  |  |  |  |  |
| Very good | 37.49% | 60.00% | 38.58% | <0.001 |  |
| Good | 47.94% | 22.22% | 46.70% |  |  |
| Average | 14.57% | 17.78% | 14.72% |  |  |
| **Have been annoyed** |  |  |  |  |  |
| Yes | 18.46% | 33.33% | 19.18% | 0.001 |  |
| No | 81.54% | 66.67% | 80.82% |  |  |
| **Percentage of vaccine refusals** |  |  |  |  |  |
| Never chose to refuse the vaccine | 64.71% | 0.00% | 61.55% | <0.001 |  |
| Chose to refuse the vaccine 1 to 3 times | 20.71% | 0.00% | 19.70% |  |  |
| Chose to refuse the vaccine 4 to 7 times | 10.33% | 0.00% | 9.82% |  |  |
| Chose to refuse the vaccine 8 to 11 times | 4.24% | 0.00% | 4.04% |  |  |
| Always chose to refuse the vaccine | 0.00% | 100.00% | 4.89% |  |  |
|  |  |  |  |  |  |
| Failed to the rationality test | 5.52% | 0.00% | 5.52% | 0.017 |  |
| Failed to the temporal consistency test | 24.16% | 0.00% | 24.16% | <0.001 |  |
|  |  |  |  |  |  |

^1^The $p$-values refer to tests between groups using one-way analysis of variance, Kruskal-Wallis H test, Bartlett’s test for equality of variances, Fisher's exact test, and Chi2 test of independence.

^2^The choice certainty score corresponds to the sum of each twelve certitude questions "Are you sure of your choice?" scaled between 0 to 10. The score is thus ranging from 0 to 120.

# **Supplementary File 7** – Multinomial Mixed Logit model interpretation

| **Attribute** | **Mean coefficient (**$\boldsymbol{b}_{\boldsymbol{k}}$**)** | **Standard deviation coefficient (**$\boldsymbol{s}_{\boldsymbol{k}}$**)** | $\boldsymbol{-}\frac{\boldsymbol{b}_{\boldsymbol{k}}}{\boldsymbol{s}_{\boldsymbol{k}}}$ | **Standard normal distribution** | **1- standard normal distribution** |
| --- | --- | --- | --- | --- | --- |
|  |  |  |  |  |  |
| **ASC** | 6.294 | 4.332 | -1.453 | 7.31% | 92.69% |
|  |  |  |  |  |  |
| **Type of vaccine** |  |  |  |  |  |
| Viral vector vaccine | (reference) | (reference) |  |  |  |
| mRNA vaccine | 0.035 | 0.393 | -0.089 | 46.47% | 53.53% |
| Subunit vaccine | -0.082 | 0.442 | 0.185 | 57.35% | 42.65% |
| DNA vaccine | -0.094 | 0.224 | 0.421 | 66.33% | 33.67% |
| Inactivated vaccine | 0.038 | 0.597 | -0.064 | 47.47% | 52.53% |
| Live-attenuated vaccine | -0.116 | 0.398 | 0.290 | 61.42% | 38.58% |
| **Vaccine effectiveness** |  |  |  |  |  |
| 95% | (reference) | (reference) |  |  |  |
| 85% | -0.656 | 0.153 | 4.281 | 100.00% | 0.00% |
| 75% | -1.380 | 0.393 | 3.512 | 99.98% | 0.02% |
| 60% | -2.563 | 1.379 | 1.858 | 96.84% | 3.16% |
| **Side effects of the vaccine** |  |  |  |  |  |
| None | (reference) | (reference) |  |  |  |
| 1 in 1,000 chances of having a minor effect | -0.536 | 0.369 | 1.453 | 92.69% | 7.31% |
| 1 in 10,000 chances of having a major effect | -1.414 | 1.411 | 1.002 | 84.19% | 15.81% |
| **Vaccination location** |  |  |  |  |  |
| A drugstore | (reference) |  |  |  |  |
| A care center | 0.003 | 0.170 | -0.017 | 49.31% | 50.69% |
| A sports center, an exhibition center | -0.023 | 0.306 | 0.075 | 52.97% | 47.03% |
| **Priority population to receive the vaccine** | | | | | |
| No priority population | (reference) | (reference) |  |  |  |
| Vulnerable people with a great loss of autonomy | -0.034 | 0.268 | 0.128 | 55.11% | 44.89% |
| Primary and secondary teachers | -0.251 | 0.397 | 0.631 | 73.60% | 26.40% |
| **Number of shots to be received** |  |  |  |  |  |
| 1 shot | (reference) | (reference) |  |  |  |
| 2 shots | -0.228 | 0.352 | 0.648 | 74.14% | 25.86% |
| 3 shots | -0.928 | 0.948 | 0.979 | 83.61% | 16.39% |
| **Vaccine whose results have been published in a scientific journal** | | | | | |
| Results published in an international journal | (reference) | (reference) |  |  |  |
| Results published in a national journal | -0.148 | 0.037 | 3.943 | 100.00% | 0.00% |
| Unpublished results | -1.461 | 1.325 | 1.103 | 86.49% | 13.51% |
|  |  |  |  |  |  |

Note: These numbers are given by $100\times\phi(-\frac{b_{k}}{s_{k}})$ with $\phi$ the cumulative standard normal distribution given by $\phi\left( x \right)=\frac{1}{\sqrt{2\pi}}\int_{-\infty}^{x} e^{-\frac{t^{2}}{2}}dt$, $b_{k}$ the mean coefficient of the level $k$, and $s_{k}$ the standard deviation coefficient for the level $k$ (Hole, 2007)^3^.

# **Supplementary File 8 - Latent Class Logit information criteria**

| **Number of latent classes** | **Number of parameters** | **AIC** | **CAIC** | **BIC** | **LL** | **Relative likelihood** |
| --- | --- | --- | --- | --- | --- | --- |
| Q=2 | 44 | 31,941.59 | 32,229.38 | 32,185.38 | -15,926.79 | 0.00E+00 |
| Q=3 | 69 | 30,592.18 | 31,043.49 | 30,974.49 | -15,227.09 | 0.00E+00 |
| Q=4 | 94 | 30,078.17 | 30,692.98 | 30,598.98 | -14,945.08 | 0.00E+00 |
| Q=5 | 119 | 29,350.35 | 30,128.68 | 30,009.68 | -14,556.17 | 4.03E-203 |
| Q=6 | 144 | 28,907.16 | 29,849.01 | 29,705.01 | -14,309.58 | 6.97E-107 |
| Q=7 | 169 | 28,724.13 | 29,829.49 | 29,660.49 | -14,193.06 | 3.87E-67 |
| Q=8 | 194 | 28,534.10 | 29,802.98 | 29,608.98 | -14,073.05 | 7.12E-26 |
| Q=9 | 219 | 28,418.29 | 29,850.69 | 29,631.69 | -13,990.15 | - |

# **Supplementary File 9** – Raw utility values by model (Multinomial Mixed Logit and Latent Class Logit)

# **Supplementary File 10** – Statistics of class membership posterior probabilities derived from the Latent Class Logit model

| **Class membership posterior probabilities** | **Latent Class Logit** | | | |
| --- | --- | --- | --- | --- |
|  | Class 1 | Class 2 | Class 3 | Class 4 |
| Mean | 0.198 | 0.553 | 0.176 | 0.074 |
| Standard deviation | 0.371 | 0.445 | 0.311 | 0.257 |
| Range | (0.000-0.999) | (0.000-0.999) | (0.000-0.999) | (0.000-0.999) |
| Median | 0.002 | 0.761 | 0.004 | 0.000 |

# **Supplementary File 11 - Mean and median response time to the 12 choice tasks**

Notes: mean response time (SD) = 05:18 (05:57) and median response time = 04:10.

# **References**

1. Ahorsu DK, Lin CY, Imani V, Saffari M, Griffiths MD, Pakpour AH. The Fear of COVID-19 Scale: Development and Initial Validation. *Int J Ment Health Addict*. Published online March 27, 2020:1-9. doi:10.1007/s11469-020-00270-8

2. Attieh R, Koffi K, Touré M, Parr-Labbé É, Pakpour AH, Poder TG. Validation of the Canadian French version of the fear of COVID-19 scale in the general population of Quebec. *Brain Behav*. Published online March 30, 2022:e32550. doi:10.1002/brb3.2550

3. Antonovsky A. The structure and properties of the sense of coherence scale. *Soc Sci Med*. 1993;36(6):725-733. doi:10.1016/0277-9536(93)90033-z

4. Schumann A, Hapke U, Meyer C, Rumpf HJ, John U. Measuring sense of coherence with only three items: a useful tool for population surveys. *Br J Health Psychol*. 2003;8(Pt 4):409-421. doi:10.1348/135910703770238275

5. Shapiro GK, Tatar O, Dube E, et al. The vaccine hesitancy scale: Psychometric properties and validation. *Vaccine*. 2018;36(5):660-667. doi:10.1016/j.vaccine.2017.12.043
